# The Effect of Lifetime Noise Exposure and Aging on Speech-Perception-in-Noise Ability and Self-Reported Hearing Symptoms: An Online Study

**DOI:** 10.3389/fnagi.2022.890010

**Published:** 2022-05-30

**Authors:** Adnan M. Shehabi, Garreth Prendergast, Hannah Guest, Christopher J. Plack

**Affiliations:** ^1^Manchester Centre for Audiology and Deafness, University of Manchester, Manchester, United Kingdom; ^2^Department of Audiology and Speech Therapy, Birzeit University, Birzeit, Palestine; ^3^Department of Psychology, Lancaster University, Lancaster, United Kingdom

**Keywords:** noise exposure, aging, cochlear synaptopathy (CS), age-related hearing loss (ARHL), speech perception in noise (SPiN), self-reported hearing, tinnitus, hyperacusis

## Abstract

Animal research shows that aging and excessive noise exposure damage cochlear outer hair cells, inner hair cells, and the synapses connecting inner hair cells with the auditory nerve. This may translate into auditory symptoms such as difficulty understanding speech in noise, tinnitus, and hyperacusis. The current study, using a novel online approach, assessed and quantified the effects of lifetime noise exposure and aging on (i) speech-perception-in-noise (SPiN) thresholds, (ii) self-reported hearing ability, and (iii) the presence of tinnitus. Secondary aims involved documenting the effects of lifetime noise exposure and aging on tinnitus handicap and the severity of hyperacusis. Two hundred and ninety-four adults with no past diagnosis of hearing or memory impairments were recruited online. Participants were assigned into two groups: 217 “young” (age range: 18–35 years, females: 151) and 77 “older” (age range: 50–70 years, females: 50). Participants completed a set of online instruments including an otologic health and demographic questionnaire, a dementia screening tool, forward and backward digit span tests, a noise exposure questionnaire, the Khalfa hyperacusis questionnaire, the short-form of the Speech, Spatial, and Qualities of Hearing scale, the Tinnitus Handicap Inventory, a digits-in-noise test, and a Coordinate Response Measure speech-perception test. Analyses controlled for sex and cognitive function as reflected by the digit span. A detailed protocol was pre-registered, to guard against “p-hacking” of this extensive dataset. Lifetime noise exposure did not predict SPiN thresholds, self-reported hearing ability, or tinnitus handicap in either age group. Exploratory analyses showed that worse hyperacusis scores, and a greater prevalence of tinnitus, were associated significantly with high lifetime noise exposure in the young, but not in the older group. Age was a significant predictor of SPiN thresholds and the presence of tinnitus, but not of self-reported hearing ability, tinnitus handicap, or severity of hyperacusis. Consistent with several lab studies, our online-derived data suggest that older adults with no diagnosis of hearing impairment have a poorer SPiN ability and a higher risk of tinnitus than their younger counterparts. Moreover, lifetime noise exposure may increase the risk of tinnitus and the severity of hyperacusis in young adults with no diagnosis of hearing impairment.

## Introduction

Presbycusis, which is also known as age-related hearing loss (ARHL), is a common condition in older adults caused by a combination of factors including lifetime cumulative noise exposure, genetic susceptibility, metabolic changes in the cochlea, and intake of ototoxic substances (Gordon-Salant et al., 2010). Excessive noise exposure and aging, as two independent factors, are associated with damage to the outer hair cells, the inner hair cells, and the spiral ganglion cells (Wang et al., 2002; Gates and Mills, 2005; Sergeyenko et al., 2013; Jayakody et al., 2018). This damage often results in deterioration of hearing sensitivity, loss of frequency selectivity, and poorer temporal resolution (Ashmore et al., 2010).

The cochlear synapses which connect inner hair cells with afferent auditory nerve fibers (ANFs) have been shown to degenerate in several animal species because of acoustic over-exposure and aging, well before outer and inner hair cells are lost. Thus, this cochlear synaptopathy (CS) can take place in the absence of hearing threshold elevation in the standard audiometric range (Kujawa and Liberman, 2009; Lin et al., 2011; Makary et al., 2011; Furman et al., 2013; Sergeyenko et al., 2013; Viana et al., 2015; Valero et al., 2017; Hickman et al., 2018; Parthasarathy and Kujawa, 2018; Wu et al., 2019; Fernandez et al., 2020). However, CS may be accompanied by permanent threshold elevations at the highest frequencies of the hearing range, which reflect the extreme basal cochlear regions (Hickox et al., 2017). Post-mortem human temporal bone data confirm an age-related synapse and ANF loss in older adults with no otologic symptoms (Viana et al., 2015; Wu et al., 2019, 2021). Noise exposure and aging seem to preferentially affect low-to-medium-spontaneous-rate high-threshold ANFs in some rodents (Schmiedt et al., 1996; Furman et al., 2013) though not in other species (Suthakar and Liberman, 2021).

It is suggested that the loss of low-to-medium spontaneous rate ANFs due to CS results in poorer temporal resolution for moderate-to-high-level acoustic stimuli such as speech (Kujawa and Liberman, 2009; Sergeyenko et al., 2013; Bharadwaj et al., 2014; Shaheen et al., 2015; Fernandez et al., 2020). Several human studies have investigated the effects of noise exposure and aging on speech-perception-in-noise (SPiN) thresholds. The majority of studies that investigated young normal-hearing humans with extensive noise exposure have failed to establish an association between lifetime noise exposure and SPiN performance (for reviews see Bramhall et al., 2019 and Le Prell, 2019). In contrast, several studies have documented higher (i.e., worse) SPiN thresholds among older adults with normal/near-normal audiograms compared to their younger counterparts (Pichora-Fuller et al., 1995; Kim et al., 2006; Füllgrabe et al., 2015; Vermeire et al., 2016; Babkoff and Fostick, 2017; Patro et al., 2021). However, it is worth highlighting that those studies did not attempt to isolate the effects of CS on SPiN performance from other factors which can potentially affect SPiN at an older age, such as central auditory neural degeneration (which may decrease temporal resolution; Caspary et al., 2008; Ouda et al., 2015), poorer cognitive function (Humes and Dubno, 2009; Kamerer et al., 2019), and worse extended high-frequency thresholds (Stelrnachowicz et al., 1989; Snell et al., 2002). The current study attempted to isolate the effects of auditory factors such as CS on SPiN ability by employing a cognitive task to control for the effects of age-related central and cognitive decline with regards to SPiN performance.

Johannesen et al. (2019) investigated the effects of noise exposure and aging in audiometrically normal adults (*n* = 94) aged 18–68 years using sentences from the hearing in noise test fixed at 65 dB SPL and disyllabic words at 50-, 65-, and 75-dB SPL. The masking noise was changed adaptively and was either speech-shaped noise or the international female fluctuating masker. Hearing-in-noise-test thresholds using both the speech-shaped-noise and the international female fluctuating maskers were significantly worse for older adults. No effects of age were evident on disyllabic words when combined across different speech levels, using either masker. The authors reported no interaction between age and the speech presentation levels in relation to SRTs obtained using disyllabic words.

Prendergast et al. (2019) and Carcagno and Plack (2021) studied the effects of various lifetime noise exposures (i.e., occupational, recreational) and age in audiometrically normal/near normal adults (*n* = 156 and *n* = 102, respectively) using the Coordinate Response Measure (CRM) speech task and the Digits In Noise (DIN) test. Carcagno and Plack (2021) employed low-pass-filtered speech stimuli (with a cut-off frequency of 3 kHz) in both SPiN tasks. CRM Stimuli were presented at low (i.e., 39 dB SPL) and high (i.e., 74 dB SPL) levels embedded in pink band-pass filtered noise (3–8 kHz) to reduce the contribution of basal cochlear generators. Prendergast et al. (2019) presented both the CRM (embedded in competing speech utterances) and DIN (embedded in speech-shaped noise of bandwidth of 0–10 kHz) stimuli at 40- and 80-dB SPL. Neither study found a significant effect of either lifetime noise exposure or age on the CRM thresholds (at either level). However, Prendergast et al. (2019) reported that older age was unexpectedly associated with better DIN thresholds at low stimulus levels while higher lifetime noise exposure was associated with better thresholds at high stimulus levels. In contrast, Carcagno and Plack (2021) found that neither age nor noise exposure had effects on DIN thresholds using band-limited stimuli. In their secondary analyses, Prendergast et al. (2019) found that the 16 kHz absolute threshold was a significant predictor of DIN scores.

Banh et al. (2012) and Carcagno and Plack (2021) studied the effect of aging on self-reported hearing ability using the SSQ12 and SSQ questionnaires, respectively (*n* = 96 and *n* = 102, respectively). Both studies found that older age is not associated with worse SSQ12 scores in audiometrically normal older adults (as defined by normal hearing thresholds up to 4 kHz). However, Banh et al. (2012) found that older adults with moderate sensorineural hearing loss exhibited significantly worse SSQ12 scores compared to their younger and older audiometrically-normal counterparts.

Both noise exposure and aging are well-established risk factors for tinnitus and hyperacusis (Gates and Mills, 2005; Kim et al., 2015; Paulin et al., 2016; Oosterloo et al., 2021). According to the British Tinnitus Association, tinnitus is defined as “*The perception of sound in the absence of any corresponding external sound. This noise may be heard in one ear, in both ears, in the middle of the head, or it may be difficult to pinpoint its exact location. The noise may be low, medium, or high-pitched. There may be a single noise or two or more components. The noise may be continuous, or it may come and go*” (Mancktelow, 2022). Hyperacusis is defined as pathological intolerance and hypersensitivity to moderate sounds (Tyler et al., 2014).

The effects of both tinnitus and hyperacusis are commonly quantified both clinically and for research purposes using self-reported psychometrically validated questionnaires such as the tinnitus handicap inventory (THI; Newman et al., 1996) and the Khalfa hyperacusis questionnaire (Khalfa et al., 2002). The THI is composed of 25 questions that evaluate the severity and impact of tinnitus on the participant’s daily life functioning (Newman et al., 1996). The total maximum score of the THI is 100 points, with a higher score reflecting a more severe tinnitus handicap (Newman et al., 1996). The Khalfa hyperacusis questionnaire consists of 14 questions that investigate intolerance to sounds through common daily life settings (Khalfa et al., 2002). Each question is scored on a 4-point scale with 1 point reflecting almost no hypersensitivity to moderate sounds and 4 points corresponding to the maximum intolerance to moderate sounds (Khalfa et al., 2002).

Some studies have quantified the effect of lifetime noise exposure on the presence of tinnitus and its handicap and on hyperacusis. For instance, Guest et al. (2017) compared the lifetime noise exposure of two groups of young audiometrically-normal hearing groups: a tinnitus group (*n* = 20) and a control group (*n* = 20). The authors found that the tinnitus group exhibited significantly higher lifetime noise exposure compared to the control group. Couth et al. (2020) employed the THI and the Khalfa hyperacusis questionnaire to compare tinnitus handicap and hyperacusis severity, respectively, in two groups of young audiometrically-normal adults: musicians (*n* = 85) and non-musicians (*n* = 52). Although the mean THI scores in both groups corresponded to the no handicap/slight handicap category, the mean THI score of musicians was significantly higher than that of non-musicians. Similarly, the authors found that musicians exhibited significantly higher (i.e., worse) hyperacusis scores compared to their non-musician counterparts. Yilmaz et al. (2017) quantified the severity of hyperacusis using the Turkish version of the Khalfa hyperacusis questionnaire among 536 university students. Although this study did not quantify noise exposure, nor did it quantify the hearing status of participants, students who reported being regularly exposed to loud noises had significantly higher hyperacusis scores compared to those without self-report of excessive noise exposure.

The current study, which employed novel online instruments to collect both behavioral and self-report hearing data remotely, aimed to assess and quantify the effects of lifetime noise exposure and aging on the hearing ability of older adults in the United Kingdom (UK). It is worth highlighting that this online study, unlike the majority of previous studies in the literature, was not limited to a sample of participants who were able and willing to attend a laboratory but rather targeted a broader UK demographic that may be exposed to a variety of noise sources and may be composed of various ethnic and socio-cultural backgrounds. The study compared the effects of both noise and aging on (i) SPIN thresholds using an online DIN test, (ii) self-reported hearing ability, and (iii) the presence of tinnitus. In exploratory data analyses, we determined the effects of lifetime noise exposure and aging on (i) the SPiN thresholds using an online version of the CRM test, (ii) tinnitus presence and handicap, and (iii) the severity of hyperacusis. We hypothesized that higher lifetime noise exposure and older age would be associated with (i) higher SPiN thresholds, (ii) worse self-reported hearing ability, (iii) a higher proportion of participants with tinnitus, (iv) worse tinnitus handicap, and (v) greater severity of hyperacusis. We found no evidence for poorer SPiN performance, worse self-reported hearing, or higher severity of tinnitus handicap as a function of higher lifetime noise exposure in either age group. Higher prevalence of tinnitus and greater severity of hyperacusis were associated with higher lifetime noise exposure in the young, but not in the older, group. Finally, aging was associated with poorer SPiN performance and a higher prevalence of tinnitus.

## Materials and Methods

This study was pre-registered on the Open Science Framework before the beginning of the data collection as part of a larger lab-based research project. Due to the COVID-19 pandemic, the original lab-based study plan was changed, and an amendment was put in place to reflect the intention to collect data online before the actual data collection started. All the hypotheses, data collection procedures, and primary statistical analyses of the current online study are in line with the pre-registered protocol as shown in the amended document.^1^

### Participants

A total of 295 adult participants for whom English was either their native (*n* = 227) or second language (*n* = 68) were recruited into two age groups (“young” *n* = 217, 151 females, age range: 18–35 years, mean age: 24.6 years; and “older” *n* = 78, 50 females, age range: 50–70 years, mean age: 58.0 years) through online advertising including social media, the University of Manchester research volunteering platform, and various young and older adult charities and societies in the United Kingdom.

Participants reported no diagnosis of hearing loss, current middle-ear pathologies, past ear surgeries, head trauma, ototoxic exposure, neurological disorders, or past diagnosis of cognitive impairments. Forty-eight participants were excluded due to reporting a past diagnosis of hearing impairments (*n* = 15), a suspicion of (based on the AD8 dementia screening tool) or past diagnosis of cognitive/memory impairment (*n* = 14), or being in an age group that did not match the inclusion criteria of the current study (*n* = 19).

In order to test the effect of age, independent of noise exposure, on the different primary and secondary outcome measures, participants in both age groups with low lifetime noise exposure (as defined below in section “Statistical Analyses”) were allocated into low-noise groups. A total of 175 participants (mean age = 24.6, females = 122) and 56 participants (mean age = 58.0, females = 34) formed the young and older low noise groups, respectively.

Upon participation, participants provided their written informed consent online for taking part in the study. A prize draw was offered as an incentive to participants. The study procedures were approved by the University of Manchester Research Ethics Committee (ethics application reference: 2020-8884-13533).

### Online Instruments

This study was carried out entirely using the Research Electronic Data Capture (REDCap) platform hosted at the University of Manchester (Harris et al., 2009, 2019). REDCap allows online research data collection through a secured electronic platform that allows data validation, integration, manipulation, and export to different statistical packages. All 295 participants completed all the online instruments, except for the DIN test, CRM task, and THI questionnaire. All native English participants were invited to perform the SPiN tasks. However, a subset of them (141 participants; 62% of native English participants; 48% of the total sample) completed the DIN and CRM tasks. Since 67 participants out of 295 (23% of the total sample) reported tinnitus, the THI questionnaire was performed by 67 participants only.

#### Otologic Health and Demographic Information

The clinical and demographic online questionnaire (see Supplementary Material) was used to collect relevant demographic as well as hearing and general health information. Demographic questions covered participants’ age, sex, educational attainment, and contact details. Participants were asked about their otologic and hearing health histories such as past ear/hearing disorders or surgeries, family history of hearing impairment, tinnitus, hyperacusis, balance problems, and intake of ototoxic drugs. Moreover, participants were asked to identify any past or current chronic health conditions and/or disabilities and subsequent intake of medications.

#### Dementia Screening

Since this study measured both self-reported hearing ability and SPiN in older adults as outcome measures, it was essential to minimize differences in performance due to central cognitive factors. The Alzheimer’s Disease (AD8) dementia screening tool (see Supplementary Material) was hence used in the form of an online self-reported questionnaire to screen for mild cognitive decline secondary to dementia. This dementia screening test is considered a time-efficient, valid, and reliable tool with high sensitivity and specificity (Galvin et al., 2005; James et al., 2006). The AD8 dementia screening online questionnaire is composed of eight statements describing different executive cognitive functions related to everyday activities (e.g., the ability to recall the correct month/year). Participants judged whether they think there has been a negative change to each of the relevant abilities. Participants with suspected dementia, based on AD8 answers, were excluded from the study and were encouraged to seek specialist advice.

#### Noise Exposure

Lifetime noise exposure was estimated using an online questionnaire based on the Noise Exposure Structured Interview (Guest et al., 2018a). This approach is based on the work of Lutman et al. (2008) and has been frequently used in recent years in some CS research studies (Guest et al., 2017, 2018b; Prendergast et al., 2017a,b, 2018, 2019; Causon et al., 2020; Couth et al., 2020; Shehorn et al., 2020). The noise exposure questionnaire (see Supplementary Material) is composed of four sections: occupational noise, recreational noise, firearm noise, and earphone/headphone noise exposure. In each section, activities that constitute potentially unsafe noise exposure (i.e., noise levels > 80 dBA) were selected/identified. For each noise exposure activity, participants then estimated the vocal effort required to converse in the selected situation, or the volume control level in the case of noise exposure from personal listening devices. These values were used to estimate the sound pressure level in dBA (Lutman et al., 2008; Guest et al., 2018a). Then participants specified the number of years, weeks per year, days per week, and hours per day of exposure for each selected activity. Finally, participants stated whether hearing protection was used for each of the specified activities and selected their type (if used). For each activity, the magnitude of lifetime noise exposure was determined by applying the following formula:


$$U=10^{\left(L-A-90\right)/10}\times \frac{T}{2080}$$

Where U = units of noise exposure (energy); L = level (dBA); A = attenuation of ear protection; T = total exposure time. The results were summed across activities to give total units of noise exposure for each participant. Participants with 0 units of lifetime noise exposure were assigned a value of 0.00001. One raw unit of noise emission (U) equates to continuous workplace exposure of 90 dB(A) for one entire working year (2,080 h). For primary and secondary data analysis purposes, the units of lifetime noise exposure were log-transformed [log_10_(U)] to produce a normally distributed variable. Hence, one logarithmic unit is equivalent to a factor of 10 in terms of lifetime noise exposure energy.

#### Cognitive Function

The digit span test with its forward and backward versions (Wechler, 1997) was incorporated as an online tool on REDCap as a measure of attention and short-term memory span. Numbers in both versions of the digit span test were presented visually on screen in an animated sequence, starting at two digits for a trial run, with each digit appearing for 1 s, and with a delay of 1 s between digits. The actual test began at two digits with the same temporal characteristics as for the trial run. For the forward digit span test, participants entered the same sequence of digits they saw (e.g., sequence: 3 2 7, correct answer: 3 2 7). For the backward digit span test, participants inputted the reverse sequence of digits (e.g., sequence: 3 2 7, correct answer: 7 2 3).

Each correct answer (all digits reported in the correct order) led to a new number sequence with an additional digit, up to a maximum of nine digits. If the entered answer was incorrect, an alternative sequence of numbers was given with the same number of digits. If the answer was incorrect twice for the same number of digits, the test ceased. The highest number of digits correctly identified was counted as the participant’s score on both the forward and backward digit span tests.

#### Hyperacusis

The degree of sensitivity and intolerance to sounds was evaluated using the Khalfa hyperacusis questionnaire (see Supplementary Material), which consists of 14 items (Khalfa et al., 2002). The items cover attentional, social, and emotional aspects related to sound intolerance. Participants judged each statement using a four-point scale such that a “no” answer corresponds to 0 points, “yes, a little” corresponds to 1 point, “yes, quite a lot” corresponds to 2 points, and “yes, a lot” corresponds to 3 points. The scores of all statements were added (per subject) and a mean score (out of 4 points) was calculated per participant.

#### Self-Reported Hearing Ability

In order to evaluate participants’ subjective hearing ability in real-world situations, the short form of the Speech, Spatial, and Qualities of Hearing scale (SSQ12) was used (Noble et al., 2013; see Supplementary Material). The SSQ12 questionnaire is composed of five statements from the speech domain, three statements from the spatial domain, and four statements from the qualities of the hearing domain. The SSQ12 was chosen instead of the full version SSQ in this study since it is faster to complete and may have adequate validity, reliability, and sensitivity compared to the full version of the SSQ (Noble et al., 2013; Ou and Kim, 2017).

Participants were asked to rate each statement of the SSQ12 using a 0–10 scale such that a higher score represents better performance. If a statement does not apply to a participant, they can select the “not applicable” option. A mean SSQ12 score was calculated per participant based on all applicable statements that they rated (non-applicable statements were unscored).

#### Tinnitus Handicap

The THI was used to assess the severity and impact of tinnitus on the participant’s daily life functioning. The THI (see Supplementary Material) is a standardized self-reported questionnaire that is composed of 25 questions that cover the potential physical, psychological, social, emotional, and occupational impact of tinnitus (Newman et al., 1996). Participants were asked whether they currently suffer from tinnitus, defined by the British Tinnitus Association (Mancktelow, 2022) as “*The perception of sound in the absence of any corresponding external sound. This noise may be heard in one ear, in both ears, in the middle of the head, or it may be difficult to pinpoint its exact location. The noise may be low, medium, or high-pitched. There may be a single noise or two or more components. The noise may be continuous, or it may come and go*.”

Participants who reported suffering from tinnitus were asked to answer each question of the THI by judging the occurrence of each situation by either choosing one of the following choices “*Always*,” “*Sometimes*,” or “*Never*.” Statements answered with “*Always*” were allocated 4 points, whereas those answered with “*Sometimes*” received 2 points. Statements answered with “*Never*” were given 0 points. The THI score was then determined per participant by summing the corresponding scores across questions.

#### Speech-in-Noise Tasks

Participants completed the DIN and CRM tests *via* a custom-designed web page, mouse/trackball or trackpad, and their own headphones or earphones. The DIN was used as the primary outcome measure to determine SPiN ability, while the CRM result was used for exploratory analyses. Both the SPiN tasks are comprised of closed-set, simple, familiar words, limiting the effects of some linguistic factors which could compromise sensitivity to synaptopathy-related SPiN deficits (Guest et al., 2018b).

Since participants performed both tasks individually, it was important to ensure that presentation levels were comfortably within the dynamic range of hearing, so that (a) performance was not limited by the audibility of the target speech and (b) stimuli were not uncomfortably loud. Participants completed an initial subjective calibration phase, involving speech stimuli presented at two sound levels, separated by 25 dB. They were instructed to adjust the volume control on their computers so that the low-level speech was clear, and the high-level speech was loud but not uncomfortable. Subsequent test stimuli were presented at an RMS level 20 dB above that of the low-level calibration stimulus and 5 dB below that of the high-level calibration stimulus. Since threshold signal-to-noise ratios were unlikely to ever fall below −20 dB, this was designed to ensure that, during testing, target speech did not become inaudible, even at low signal-to-noise ratios.

Participants were instructed to run both tests in a quiet room, away from distractions, using their personal computers or laptops (i.e., not smartphones or tablets), and to listen to the tasks through their headphones/earphones (i.e., not built-in speakers). Each listening task involved one training block which lasted about 4 min and one 5-min testing block. In order to maintain the participant’s attention and engagement, visual feedback was presented following each trial, showing whether the subject’s response was correct or incorrect and the difficulty level they reached at each trial.

##### Online Digits-in-Noise Test

Thresholds for the DIN task are thought to be primarily determined by the health of the peripheral auditory system rather than by central factors (Heinrich et al., 2015). Therefore, this task may help to capture peripheral auditory factors associated with SPiN ability by minimizing variability due to cognitive factors. Moreover, Heinrich et al. (2015) showed that performance on the DIN task may be associated with self-reported auditory function using the SSQ tool, which suggests that it may reflect a realistic representation of real-life listening abilities. Hence, the DIN was used as the primary measure of SPiN ability in this study.

DIN target phrases involve a carrier phrase and three digits ranging from 1 to 9 (“*The digits {digit 1} {digit 2} {digit 3}*”), embedded in speech-shaped background noise (Smits et al., 2004). The digits and background noise were low-pass filtered at a knee-point of 8 kHz to limit the effects on the performance of differences in the high-frequency responses of participants’ headphones/earphones. Stimuli were presented diotically and were spoken in English by a British female speaker. Participants were instructed to enter the three digits on a numeric keypad on the web interface. A response was counted as correct if participants entered 2/3 or 3/3 digits correctly. The signal-to-noise ratio was varied using a two-down, one-up adaptive rule, with four initial turn points (6 dB step size) and six subsequent turn points (2 dB step size). The signal-to-noise ratios at the final six turn points was averaged to yield the threshold. Before each of the actual scored DIN and CRM tasks began, participants performed short (4-min) practice blocks for both tests.

##### Online Coordinate Response Measure Speech Test

The CRM test involves phrases of the form “*Ready {call-sign}, go to {color} {number} now*” (Bolia et al., 2000), articulated by four male and four female British-English talkers (Kitterick et al., 2010). The stimulus library consisted of eight call signs, four colors (green, red, blue, and white), and four numbers (1, 2, 3, and 4). The target speech was a randomly selected phrase with the call-sign “*Baron*.” The competing speech was composed of two simultaneously presented phrases, spoken by the same or a different talker, each randomly selected from the library excluding the call-sign “*Baron*.” As with the DIN test, the CRM speech stimuli and the masker were low-pass filtered at a knee-point of 8 kHz. The phrases were spatialized by convolving them with head-related impulse responses so that the target was presented at 0° and the two maskers at −60° and + 60°. Participants were instructed to identify the color and number associated with the call-sign “*Baron*” only, entering them *via* mouse and browser in a 16-alternative, forced-choice paradigm.

During the CRM test, the signal-to-noise ratio was varied using an adaptive two-down, one-up stepping rule with a 6-dB step size for the first four turnpoints and a 2-dB step size for the final six turnpoints. The threshold was estimated by taking an average of the signal-to-noise ratio at the final six turnpoints.

### Statistical Analyses

Data were analyzed using the statistical package for social sciences (SPSS) version 26 software, while figures were generated using R (R Core Team, 2020). The main analyses determined the effects of lifetime noise exposure (in the older group) and age (in the low-noise groups) on (i) self-reported hearing ability, (ii) the presence of tinnitus, and (iii) SPiN performance as reflected by DIN thresholds. Multiple linear regression models were used to test (i) and (iii), while logistic regression models were employed to test (ii).

For all primary and exploratory research questions, the predictor variables were lifetime noise exposure and age, while the sex of participants and their cognitive function (as reflected by the forward and backward digit span test scores) were considered covariates in all the statistical models. The effect of lifetime noise exposure on the different outcome measures in the older group was a primary focus of this study because the majority of previous studies involved young audiometrically normal adults, with only a few studies assessing the perceptual effects of noise-induced CS in older normal-hearing adults (Valderrama et al., 2018; Prendergast et al., 2019; Yeend et al., 2019; Carcagno and Plack, 2021).

In order to assess the effect of age in the primary and exploratory research questions independent of lifetime noise exposure, only participants with low lifetime noise exposure were included in these analyses. This is defined as less than 1.0 unit of lifetime noise exposure in the protocol pre-registered prior to the beginning of data collection. This criterion is consistent with the classification of Prendergast et al. (2017b) of “low” lifetime noise exposure, corresponding to the 25% lowest lifetime noise exposure scores of their cohort (*n* = 141).

Alpha level was adjusted for six multiple comparisons using the Bonferroni-Holm method, with a familywise error rate of < 0.05. Exploratory analyses were performed to test the effect of lifetime noise exposure (in the young group) and age (in the low-noise group) on (i) the severity of hyperacusis, (ii) tinnitus handicap, and (iii) SPiN performance as shown by the CRM thresholds. Multiple linear regression models were employed to test the effects of lifetime noise exposure and age on the secondary outcome variables. The covariates of sex and cognitive ability were accounted for in the multiple linear regression models of the secondary analyses.

Further exploratory multiple regression models were performed to assess the interaction between lifetime noise exposure and age on (1) DIN thresholds, (2) CRM thresholds, (3) SSQ12 scores, (4), and the proportion of participants with tinnitus, (5) the THI scores, and (6) hyperacusis scores. Both lifetime noise exposure and age were the predictor variables, while the sex of participants and their cognitive function as reflected by the forward and backward digit span scores were considered covariates.

All analyses followed the pre-registered protocol. However, to address the potential concern that participants with no reported noise exposure could exert undue influence on the statistical models, all analyses using lifetime noise exposure scores were re-run with these participants excluded. These additional analyses are reported in Supplementary Material.

## Results

### Lifetime Noise Exposure

**FIGURE 1:**
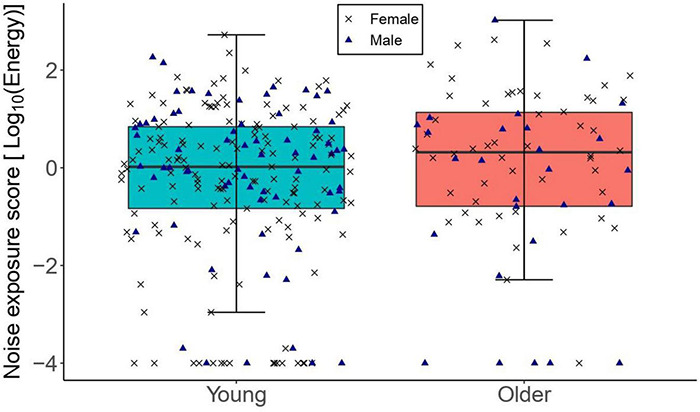
The distribution of lifetime noise exposure for the young (*n* = 217) and older (*n* = 78) groups. The left-hand side boxplot corresponds to the older group, while the right-hand side boxplot corresponds to the young group. The upper and lower hinges boxes represent the first and the third quartiles, the thick line the median, the upper whiskers the highest value within 1.5 * IQR (interquartile range) of the upper hinge, and lower whiskers the lowest value within 1.5 * IQR of the lower hinge. Black crosses and blue triangles correspond to individual female and male participants, respectively.

Figure 1 illustrates the distribution of lifetime noise exposure in both age groups. Lifetime noise exposure scores were spread similarly across the two age groups. Since lifetime noise exposure scores were not normally distributed in both the young and older groups as found by the Kolmogorov–Smirnov test (*p* < 0.05), a Wilcoxon-Mann-Whitney test was used to compare the means of lifetime noise exposure across the two age groups. Lifetime noise exposure scores of the young group (median = 0.02, inter-quartile range = 1.73) were statistically similar to those of the older group (median = 0.32, inter-quartile range = 2.0; *U* = 9410, *p* = 0.142).

### Speech Perception in Noise

#### The Effect of Lifetime Noise Exposure

**FIGURE 2:**
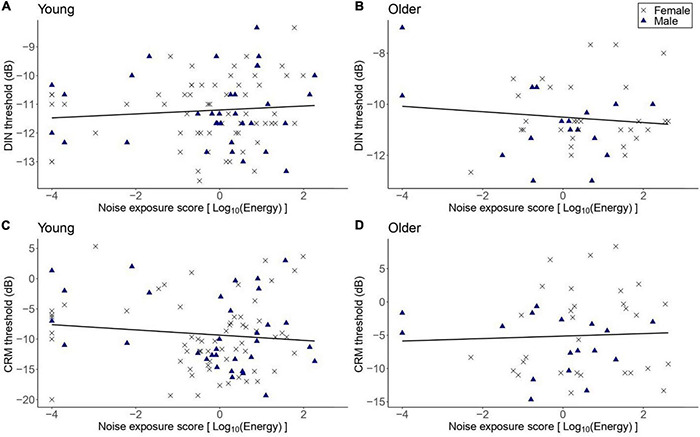
DIN and CRM thresholds as a function of lifetime noise exposure. **(A,B)** DIN and thresholds in the young (*n* = 94) and older (*n* = 47) groups, respectively. **(C,D)** CRM thresholds in the young and older groups, respectively. Black crosses and blue triangles represent individual female and male participants, respectively. Best-fit regression lines are drawn through the data points.

Figure 2 illustrates SPiN thresholds as a function of lifetime noise exposure (expressed in logarithmic units). The primary linear regression model showed that the DIN thresholds in the older group did not vary significantly as a function of lifetime noise exposure [*β* = -0.14, *t* = -0.97, *p* = 0.337]. The covariates of sex and cognitive ability (as reflected by the forward and backward digit span scores) were not significant predictors of the DIN thresholds in the older group. The exploratory multiple linear regression models showed that lifetime noise exposure did not predict the DIN thresholds in the young group [*β* = 0.05, *t* = 0.66, *p* = 0.508]; the CRM thresholds in the older group [*β* = 0.12, *t* = 0.19, *p* = 0.852]; nor the CRM thresholds in the young group [*β* = -0.04, *t* = -1.10, *p* = 0.237]. Neither sex nor cognitive function were significant predictors.

#### The Effect of Age

**FIGURE 3:**
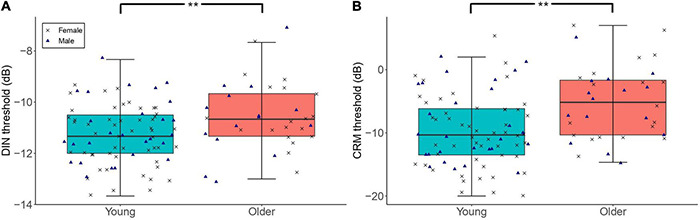
The distribution of DIN **(A)** and CRM **(B)** thresholds among participants with low lifetime noise exposure (<1.0 logarithmic units) in the young (*n* = 79) and older (*n* = 34) groups. For each of the panels, the left-hand side boxplot corresponds to the older group, while the right-hand side boxplot corresponds to the young group. The upper and lower hinges boxes represent the first and the third quartiles, the thick line the median, the upper whiskers the highest value within 1.5 * IQR (interquartile range) of the upper hinge, and lower whiskers the lowest value within 1.5 * IQR of the lower hinge. Black crosses and blue triangles correspond to individual female and male participants, respectively. ** Represent statistical outcomes with significance level < 0.01.

Figure 3 shows the DIN and CRM thresholds among participants with low lifetime noise exposure in the young and older groups. The primary linear regression model showed that the DIN thresholds were significantly higher among the low-noise older participants (mean = −10.58 dB, *SD* = 1.34 dB) compared to their young low-noise counterparts [mean = −11.26 dB, *SD* = 1.15 dB; *β* = 0.71, *t* = 2.83, *p* = 0.006]. Neither the sex of participants nor their cognitive function were significant predictors of DIN thresholds in the regression model. The exploratory regression model for the effect of age on CRM threshold showed that low-noise older participants performed significantly worse (i.e., higher thresholds; mean = −5.15 dB, *SD* = 5.81 dB) than their young low-noise counterparts [mean = −9.63 dB, *SD* = 5.64 dB; *β* = 3.97, *t* = 3.40, *p* = 0.001]. Neither sex nor cognitive function were significant predictors.

### Self-Reported Hearing Ability

#### The Effect of Lifetime Noise Exposure

**FIGURE 4:**
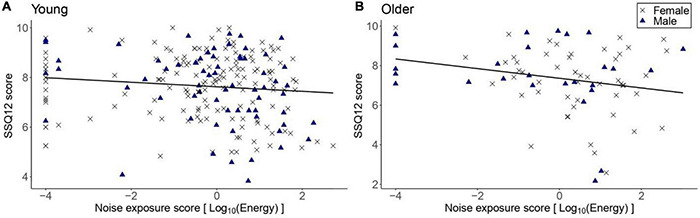
SSQ12 scores as a function of lifetime noise exposure expressed in logarithmic scale. **(A)** corresponds to the data of the young group (*n* = 217) while **(B)** to the older group (*n* = 78). Black crosses and blue triangles represent individual female and male participants, respectively. Best-fit regression lines are drawn through the data points.

Figure 4 illustrates the SSQ12 scores as a function of lifetime noise exposure. For the primary linear regression model for the older group, the SSQ12 scores did not vary significantly as a function of lifetime noise exposure [*β* = -0.24, *t* = -1.91, *p* = 0.06]. Neither sex nor cognitive function were significant predictors. For the young group, the exploratory regression model showed that lifetime noise exposure did not predict the SSQ12 scores [*β* = -0.09, *t* = -1.64, *p* = 0.104]. Neither sex nor cognitive function were significant predictors.

#### The Effect of Age

**FIGURE 5:**
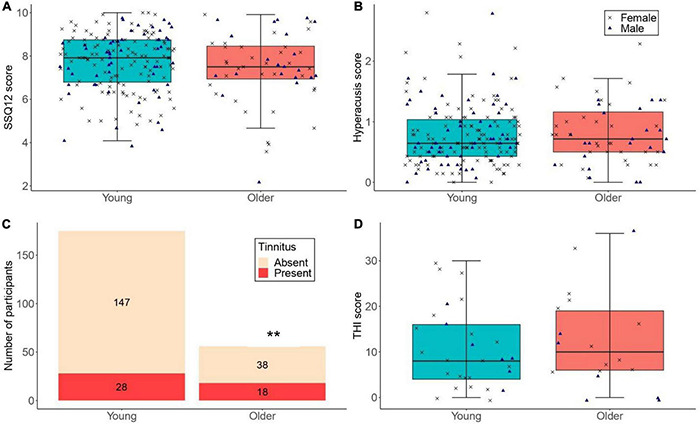
**(A,B)** The distribution of SSQ12 scores and hyperacusis scores for participants with low lifetime noise exposure (<1.0 logarithmic units) in the young (*n* = 175) and older (*n* = 56) groups. **(C)** The number of low-noise participants who reported tinnitus in the young (*n* = 175; proportion of low-noise participants with tinnitus in the young group = 16.0%) and older (*n* = 56; proportion of low-noise participants with tinnitus in the older group = 32.1%) groups (** denotes *p* < 0.01). **(D)** THI scores for low-noise participants who reported tinnitus in the young (*n* = 28) and older (*n* = 18) groups. For **(A,B,D)**, the left-hand side boxplot corresponds to the young group, while the right-hand side boxplot corresponds to the older group. The upper and lower hinges represent the first and the third quartiles, the thick line the median, the upper whiskers the highest value within 1.5 * IQR (interquartile range) of the upper hinge, and lower whiskers the lowest value within 1.5 * IQR of the lower hinge. Black crosses and blue triangles correspond to individual female and male participants, respectively.

Figure 5A shows the SSQ12 scores among low-noise participants in the young and older groups. As per the primary linear regression model, older low-noise participants had similar SSQ12 scores (mean = 7.43, *SD* = 1.64 dB) compared to their low-noise young counterparts (mean = 7.72, *SD* = 1.32) [*β* = -0.27, *t* = -1.25, *p* = 0.211]. Neither sex nor cognitive function were significant predictors.

### Tinnitus

#### The Effect of Lifetime Noise Exposure

**FIGURE 6:**
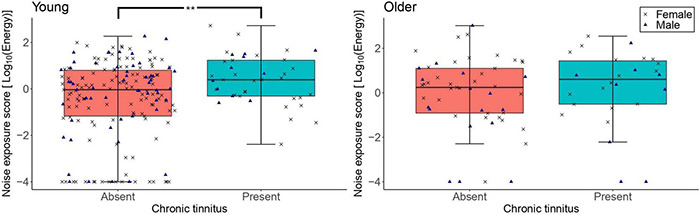
The distribution of lifetime noise exposure scores in the young (*n* = 217; left-hand-side panel) and the older (*n* = 78; right-hand-side panel) groups as a function of the presence of tinnitus. For each of the panels, the left-hand side boxplot corresponds to absent tinnitus, while the right-hand side boxplot corresponds to present tinnitus. The upper and lower hinges represent the first and the third quartiles, the thick line the median, the upper whiskers the highest value with 1.5 * IQR (interquartile range), and lower whiskers the lowest value within 1.5 * IQR of the lower hinge. Black crosses and blue triangles correspond to individual female and male participants, respectively. ** denotes *p* < 0.01.

Figure 6 shows the distribution of lifetime noise exposure scores as a function of the presence of tinnitus. The primary logistic regression model showed that lifetime noise exposure did not predict the number of participants with tinnitus in the older group (OR = 1.11, 95%CI = 0.81–1.15, *p* = 0.516). These logistic regressions are also reported in Supplementary Material, excluding participants with no noise exposure. Neither sex nor cognitive function were significant predictors. The exploratory logistic regression model for the young group showed that more participants reported tinnitus as their lifetime noise exposure increased (OR = 1.50, 95%CI = 1.13–1.99, *p* = 0.005). Neither sex nor cognitive function were significant predictors.

**FIGURE 7:**
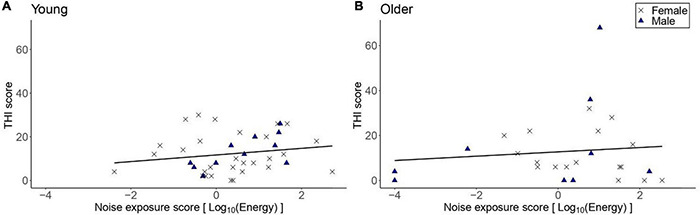
THI scores as a function of lifetime noise exposure, for those participants who reported tinnitus. **(A)** Shows the data for the young group (*n* = 40) while **(B)** the data for the older group (*n* = 26). Black crosses and blue triangles represent individual female and male participants, respectively. Best-fit regression lines are drawn through the data points.

Figure 7 shows the THI scores reported by participants with tinnitus as a function of lifetime noise exposure. Exploratory linear regression models showed that lifetime noise exposure did not predict the THI scores in the young [*β* = 1.47, *t* = 1.04, *p* = 0.307] nor older [*β* = 1.56, *t* = 0.75, *p* = 0.461] groups. Neither sex nor cognitive function were significant predictors.

#### The Effect of Age

Figure 5C illustrates the number of participants with low lifetime noise exposure who reported tinnitus. For the primary logistic regression model, the proportion of low-noise participants with tinnitus was significantly higher in the older than in the young group (OR = 2.64, 95%CI = 1.29–5.38, *p* = 0.008). Neither sex nor cognitive function were significant predictors.

Figure 5D shows the distribution of THI scores across low-noise participants who reported tinnitus in the young and older groups. The exploratory regression models showed that the THI scores were similar across low-noise participants in the young (mean = 10.57, *SD* = 8.76) and older (mean = 12.44, *SD* = 10.55) groups [*β* = 2.34, *t* = 0.77, *p* = 0.448]. Neither sex nor cognitive function were significant predictors.

### Hyperacusis

#### The Effect of Lifetime Noise Exposure

**FIGURE 8:**
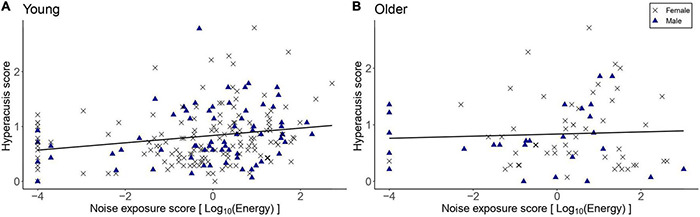
Hyperacusis scores as a function of lifetime noise exposure for the young group (*n* = 217; **A**) and the older group (*n* = 78; **B**). Black crosses and blue triangles represent individual female and male participants, respectively. Best-fit regression lines are drawn through the data points.

Figure 8 shows the hyperacusis scores as a function of lifetime noise exposure. The exploratory linear regression models showed that hyperacusis scores in the young group were significantly higher as lifetime noise exposure increased [*β* = 0.07, *t* = 3.27, *p* = 0.001]. For the older group, lifetime noise exposure did not predict hyperacusis scores [*β* = 0.01, *t* = 0.24, *p* = 0.812]. Neither sex nor cognitive function were significant predictors in either model.

#### The Effect of Age

Figure 5B shows the distribution of hyperacusis scores among low-noise participants in both age groups. The exploratory linear regression model showed that the hyperacusis scores of low-noise young participants (mean = 0.78, *SD* = 0.50) did not differ significantly from those of their older counterparts [mean = 0.82, *SD* = 0.55; *β* = 0.041, *t* = 0.510, *p* = 0.611]. Neither sex nor cognitive function were significant predictors.

### The Interaction Between Lifetime Noise Exposure and Age

In further exploratory analyses, lifetime noise exposure, age group (i.e., young and older), and an interaction term (lifetime noise exposure × age group) were included as predictors in the model for each outcome variable, with covariates of sex and cognitive function. Main effects of (1) age group on DIN thresholds [adjusted *R*^2^ = 0.13, *F*_(1)_ = 10.25, *p* = 0.013], (2) lifetime noise exposure on hyperacusis scores [adjusted *R*^2^ = 0.44, *F*_(252)_ = 1.88, *p* = 0.019], and (3) age group on the proportion of subjects with tinnitus (beta = −0.088, *p* = 0.004) were observed. The interaction between lifetime noise exposure and age group was significant for hyperacusis scores only [*F*_(1, 252)_ = 2.66, *p* = 0.034, η^2^ρ = 0.34) such that the effect of noise exposure on hyperacusis scores was smaller for the older group compared to the young. No other effects were significant.

## Discussion

We hypothesized that higher lifetime noise exposure in the older group and age (independent of noise exposure) are associated with (i) higher SPiN thresholds using the DIN test, (ii) lower SSQ12 self-reported hearing scores, and (iii) a higher proportion of participants with tinnitus. In the older group, no significant effects of lifetime noise exposure on any of our primary outcome measures were found. Older low-noise participants exhibited significantly higher DIN thresholds and a higher proportion of tinnitus than their young counterparts. Both young and older low-noise participants had similar scores on the SSQ12.

In our exploratory analyses, we examined the effects of lifetime noise exposure in the young group and age (independent of noise exposure) on (i) CRM SPiN thresholds, (ii) the number of participants with tinnitus, (iii) tinnitus handicap severity (of participants with tinnitus), and (iv) the severity of hyperacusis. In the young group, higher lifetime noise exposure was associated with a significantly greater proportion of participants with tinnitus and higher hyperacusis scores. It should be noted that there were a greater number of younger participants than older participants and so it is possible that the effect persists in the older group, but that our study had insufficient power to demonstrate it. Lifetime noise exposure did not predict performance on the CRM task. Older low-noise participants exhibited significantly higher CRM thresholds but similar hyperacusis scores compared to their young low-noise counterparts.

### Speech Perception in Noise

#### The Effect of Noise Exposure on Speech-Perception-in-Noise

SPiN thresholds obtained by both the DIN and CRM tests were similar across various lifetime noise exposure magnitudes in both the young and older groups. Although we expected differences in SPiN thresholds due to lifetime noise exposure which could potentially damage the inner ear structures which play a role in the audibility (i.e., outer hair cells) and intelligibility (i.e., inner hair cells and cochlear synapses) of speech signals at moderately loud suprathreshold levels, these findings are in line with several past studies which failed to document an effect of noise exposure on SPiN performance among normal-hearing young (Grose et al., 2017; Prendergast et al., 2017b; Yeend et al., 2017; Guest et al., 2018b; Couth et al., 2020) and older adults (Valderrama et al., 2018; Prendergast et al., 2019; Carcagno and Plack, 2021).

These negative findings could be explained by two different scenarios. First, the magnitude of cumulative lifetime noise to which participants in this study have been exposed may not have been sufficient to induce widespread hair cell and synapse loss which would translate into noticeable SPiN differences. A higher magnitude of lifetime noise exposure may therefore be necessary to induce significant SPiN effects. Moreover, it is possible that noise-induced CS may not preferentially result in degraded low- and medium- spontaneous rate ANFs, which in humans are thought to exhibit high thresholds (based on animal data) and thus code moderate-to-high acoustic information such as speech (Liberman, 1978; Furman et al., 2013). In the absence of single-unit recordings in humans, it is impossible to confirm the thresholds of ANFs lost due to noise exposure. Additionally, the effects of CS on speech perception may be insignificant compared to those of a wide array of cognitive, linguistic, and attentional factors, leaving day-to-day perception largely unaffected. Thus, the assumption that noise-induced CS results in SPiN difficulties is still under debate (Hickox et al., 2017; Le Prell, 2019).

Second, the tools used in this study may lack sufficient sensitivity to detect the hypothesized effects. For instance, it is possible that a limited noise-induced synapse loss has already occurred with minimal outer hair cell loss, however, the current DIN task may not be sufficiently sensitive to show the differences in SPiN performance. According to Oxenham (2016), a synapse loss of up to 50% in humans may not necessarily be detectable using behavioral tasks. Moreover, the currently employed method to quantify noise exposure (i.e., self-reported questionnaire) may not be reliable as it is primarily based on participants’ ability to recall instances of intense acoustic over-exposure throughout the lifespan.

#### The Effect of Age on Speech-Perception-in-Noise

SPiN hearing thresholds using both the DIN and CRM tasks were significantly higher among low-noise older participants compared to their young counterparts. These findings imply a clear age-related effect on SPiN ability and are generally consistent with several past studies (Pichora-Fuller et al., 1995; Kim et al., 2006; Füllgrabe et al., 2015; Babkoff and Fostick, 2017; Johannesen et al., 2019; Patro et al., 2021).

Prendergast et al. (2019) reported worse SPiN performance among older participants using the DIN task using the 80 dB SPL stimulus condition (but not the 40 dB SPL condition). In contrast, Carcagno and Plack (2021) failed to document such an effect using the DIN test which involved pink band-pass filtered noise at 3–8 kHz and low-pass filtered speech stimuli with a cut-off at 3 kHz presented at low and high levels. For the CRM test, neither Prendergast et al. (2019) nor Carcagno and Plack (2021) found credible age-related differences in the CRM thresholds at either stimulus level.

Several factors may have contributed to the age-related effects on SPiN ability as seen in the current study. First, since this study was conducted entirely online, it was not possible to measure and control for age-related audiometric threshold elevation both within the standard and the extended audiometric ranges (which is primarily driven by ARHL). Age-related outer hair cell loss within the standard audiometric range may compromise SPiN performance (Hoben et al., 2017; Keithley, 2020). Moreover, the elevation in extended high-frequency thresholds, which is common at an older age (Valiente et al., 2014; Wang et al., 2021), has been associated with worse SPiN performance (Monson et al., 2019; Yeend et al., 2019; Zadeh et al., 2019; Hunter et al., 2020).

Second, although we attempted to control for age-related cognitive decline, which can result in poorer SPiN performance (Humes and Dubno, 2009; Kamerer et al., 2019), poorer central auditory processing, which is typically associated with aging, could account for the observed SPiN differences (Caspary et al., 2008; Ouda et al., 2015). Interestingly, the age-related difference using the CRM task was much greater than that obtained by the DIN test. This could be due to the involvement of central factors since the CRM task is more complex than the DIN test and hence may place greater demands on cognitive factors (Heinrich et al., 2015). Third, given the inability to isolate the effects of outer hair cell loss and central auditory processing in the current study, it is impossible to rule out the contribution of age-related CS and ANF loss to the poorer SPiN performance in the older group as recent human temporal bone data provided compelling evidence in support of age-related CS and ANF degeneration (Viana et al., 2015; Wu et al., 2019, 2021).

### Self-Reported Hearing Ability

#### The Effect of Noise Exposure on the Speech Spatial and Qualities of Hearing Questionnaire

Self-reported hearing ability, as assessed by the SSQ12 questionnaire, did not vary significantly as a function of lifetime noise exposure in the young or the older groups. It is worth pointing out that participants in the older group with higher noise exposures tended to have the worse self-reported hearing ability, but the effect was non-significant even before correction for multiple comparisons (*p* = 0.06). These negative findings were similar to those of other studies which investigated normal/near-normal hearing participants of various ages and failed to document poorer scores on either the full or short versions of the SSQ as a function of higher noise, such as Prendergast et al. (2017b), Yeend et al. (2017), and Carcagno and Plack (2021).

#### The Effect of Age on the Speech Spatial and Qualities of Hearing Questionnaire

Young low-noise participants produced similar SSQ12 scores to those of older low-noise participants. These findings are in line with the outcomes reported by Füllgrabe et al. (2015) and Carcagno and Plack (2021) who documented statistically similar performance on the SSQ12 and SSQ, respectively, across normal/near-normal hearing participants of different ages. Banh et al. (2012) showed a similar outcome to our study in that the average SSQ score of older adults with normal hearing up to 4 kHz was slightly worse (mean SSQ score = 7.7, *SD* = 1.2) than the scores obtained by younger normal-hearing adults (mean SSQ score = 8.8, *SD* = 1.9). However, older adults with moderate hearing loss had significantly worse mean SSQ scores of 5.5 than younger and older normal-hearing participants (as defined by normal hearing thresholds up to 4 kHz).

The lack of significant effects of lifetime noise exposure and age on SSQ12 scores in the current study could stem, at least partially, from the possibility that most of our participants may have had normal/near-normal low- and mid-frequency hearing thresholds. Thus, the effects of self-reported hearing difficulties may be largely unaffected by high-frequency hearing losses which typically are associated with excessive noise exposure and ARHL. Moreover, the SSQ12 may lack sufficient sensitivity to detect mild hearing difficulties that may result from excessive lifetime noise exposure and aging. Finally, Füllgrabe et al. (2015) argued that, due to social and cultural factors, older adults may tend to underestimate the effects of their hearing difficulties, which hence would reduce the efficacy of using self-reported questionnaires to highlight age-related hearing difficulties.

### Tinnitus

#### The Effect of Lifetime Noise Exposure on Tinnitus

As lifetime noise exposure increased, a higher proportion of participants reported tinnitus in the young, but not in the older group. Consistent with these findings, Guest et al. (2017) showed that young normal-hearing adults with tinnitus reported significantly higher noise exposure compared to a non-tinnitus audiometrically-matched control group. Other studies, which did not perform audiometric matching between tinnitus and non-tinnitus normal-hearing young participants, also reported a significant association between recreational noise exposure and tinnitus (Meyer-Bisch, 1996; Davis et al., 1998; Degeest et al., 2014). Hearing threshold elevation secondary to noise-induced hearing loss (which is primarily characterized by OHC loss) is a well-established risk factor for tinnitus (Boger et al., 2016; Paulin et al., 2016). Moreover, noise-induced ANF loss (without hair cell loss) may be associated with increased compensatory central neural activity at the level of the mid-brain, which in humans is hypothesized to translate into tinnitus (Schaette and McAlpine, 2011; Hickox and Liberman, 2014). So, the central compensatory gain theory may at least partially explain the current findings, alongside potential hair cell loss. Schaette and McAlpine (2011) framed cochlear synaptopathy and the central compensatory gain mechanism as being potentially linked, though other processes could account for their observed data. Indeed, it is important to note that there are many other proposed mechanisms of tinnitus, such as lateral inhibition (Gerken, 1996), the central noise model (Zeng, 2013), and the stochastic resonance model (Schilling et al., 2021). It is not clear how completely the central compensatory gain theory can account for all instances of tinnitus, and it is not clear how compatible the other models are with a loss of cochlear synapses.

Although we expected to see a higher proportion of participants with tinnitus as a function of lifetime noise exposure in the older group, our current findings failed to establish such a link. Similar to these outcomes, Valderrama et al. (2018) found no significant difference in lifetime noise exposure between tinnitus and non-tinnitus middle-aged normal/near-normal hearing participants. An earlier study by Sindhusake et al. (2003) showed that self-reported occupational noise exposure among older adults (aged 55+) significantly increased the relative risk of tinnitus. This study, however, found that other factors such as age-related audiometric threshold elevations and other otologic pathologies were also associated with tinnitus at an older age. Thus, based on these findings, it is difficult to establish whether tinnitus may occur in the older population as a consequence of acoustic overexposure while hearing thresholds are still within the normal/near-normal audiometric range.

A prospective 10-year cohort study that documented the incidence and risk factors of tinnitus in middle-aged and older adults found that occupational, recreational, and firearm noise exposure were not associated with a higher incidence of tinnitus (Nondahl et al., 2010). Authors attributed the lack of association to the possible decrease in the frequency and magnitude of noise exposure at older ages compared to the younger population, which may be more involved in loud and noisy recreational and occupational events.

Our exploratory analyses showed that higher lifetime noise exposure was not associated with higher tinnitus handicap in either age group. It is possible that these secondary analyses lacked sufficient statistical power to detect the hypothesized effects of noise exposure on tinnitus severity which was documented by a few studies (Tong and Yeung, 2017; Bhatt, 2018). However, it is worth highlighting that, in line with our findings, House et al. (2018) reported that THI scores were not predicted by noise exposure. Further research is therefore necessary to establish the effect of noise exposure on the severity of tinnitus handicap in different age groups.

#### The Effect of Age on Tinnitus

In the current study, we found a significantly higher proportion of participants with tinnitus among low-noise older participants compared to their young low-noise counterparts. This implies that aging, which is typically associated with peripheral and central auditory degeneration, may increase the risk of tinnitus.

A higher risk of tinnitus at an older age is well-established across the literature (Ahmad and Seidman, 2004; Nondahl et al., 2010; Shargorodsky et al., 2010; Kim et al., 2015; McCormack et al., 2016). This age-related increase in the risk of tinnitus is often attributed to ARHL, otologic pathologies, head and neck traumas, neurological disorders, and other lifestyle factors such as smoking and alcohol consumption (Ahmad and Seidman, 2004; Shargorodsky et al., 2010; Kim et al., 2015). In the current study, while we attempted to control for factors such as lifetime noise exposure, sex of participants, otologic pathologies, head and heck traumas, past diagnosis of hearing impairment, cognitive function, and intake of ototoxic medications, we cannot rule out the contribution of undiagnosed age-related threshold elevation to the higher proportion of participants of tinnitus in the older group. This is because the presence of high-frequency hearing impairment, which typically results from age-related outer hair cell loss in basal cochlear regions, is associated with an increase in the risk of tinnitus in the older population (König et al., 2006; Terao et al., 2011).

After examining the THI scores of the participants with tinnitus as a function of lifetime noise exposure in the young and the older groups, and across low-noise participants in both age groups, neither lifetime noise exposure nor age predicted the THI scores. THI scores corresponded to the slight tinnitus handicap category across most participants in both age groups, which indicates that tinnitus is only noticeable in quiet and has no impact on sleep and daily activities (McCombe et al., 2001).

Given the exploratory nature of these analyses and the fact that the number of participants was not sufficient to provide enough power to detect an effect on THI scores, it is difficult to draw firm conclusions on whether excessive lifetime noise exposure and aging result in worse tinnitus handicap. A few studies have evaluated the risk factors associated with worse tinnitus handicap as reflected by higher THI scores. These included sex (i.e., being male), psycho-emotional disorders such as depression and anxiety, noise exposure, and aging (Hiller and Goebel, 2006; Schlee et al., 2011; Milerová et al., 2013; Bhatt, 2018; House et al., 2018). However, the evidence presented by other studies challenged these associations (Pinto et al., 2010; Figueiredo et al., 2011; Udupi et al., 2013; Frederiksen et al., 2017; Ralli et al., 2017). Further research is, therefore, necessary to determine the effects of noise exposure and aging on tinnitus handicap.

### Hyperacusis

#### The Effect of Lifetime Noise Exposure on Hyperacusis

In our exploratory analyses, we found that greater hyperacusis scores (i.e., worse hyperacusis severity) were associated with higher lifetime noise exposure in the young group, but not in the older group. The effect found in the young group is in line with the findings of several studies which documented worse hyperacusis among young noise-exposed normal-hearing adults and adolescents (Widen and Erlandsson, 2004; Yilmaz et al., 2017; Camera et al., 2019; Couth et al., 2020; Fredriksson et al., 2021; Pienkowski, 2021). Although audiometric threshold elevation in the standard audiometric range is considered a primary initiating mechanism for hyperacusis (Knipper et al., 2013; Auerbach et al., 2014; Pienkowski et al., 2014), acoustic overexposure seems to induce worse hyperacusis in young normal-hearing adults. Noise-induced loss of ANFs in the absence of outer hair cell damage may contribute to worse hyperacusis due to increased central compensatory gain (Schaette and McAlpine, 2011; Hickox and Liberman, 2014). However, the evidence on the association between noise-induced hyperacusis and increased central gain is still inconclusive (Möhrle et al., 2019; Couth et al., 2020).

The lack of association between lifetime noise exposure and hyperacusis in the older group could be explained by the hypothesis that the effect of noise exposure on the peripheral neural auditory system at an older age could manifest differently than young age. Recent temporal bone data by Wu et al. (2021) established that lifetime occupational noise exposure produced more severe ANF loss in middle-aged but not in older adults. This is consistent with rodent data from Möhrle et al. (2016) who showed that middle-aged and older rats exhibited significantly less noise-induced synapse loss compared to their young counterparts. Thus, older adults may experience limited additional perceptual auditory effects such as tinnitus and hyperacusis due to noise exposure.

#### The Effect of Age on Hyperacusis

Aging, which is typically associated with cochlear hair cell and ANF loss, has been associated with the development of hyperacusis in older adults (Andersson et al., 2002; Tyler et al., 2014; Paulin et al., 2016). Other age-related comorbidities such as cardiovascular disease, psycho-emotional disorders (e.g., depression and anxiety), and neurologic conditions such as multiple sclerosis are thought to result in worse hyperacusis severity in the older population (Tyler et al., 2014). In the current study, older low-noise participants exhibited similar hyperacusis scores compared to their young low-noise counterparts. Further research, which could potentially control for age-related cochlear hair cell loss and other aging comorbidities, is necessary to disentangle the factors which increase the risk of hyperacusis at an older age.

### Strengths and Limitations

The current study employed a novel approach to collect an extensive dataset of self-reported hearing and SPiN data using online instruments. These online data-collection tools enabled access to a wide demographic of participants from various social, cultural, ethnic, and educational backgrounds in the United Kingdom. Moreover, the convenience of remote online data collection allowed the researchers to carry out the current study during the COVID-19 pandemic when in-person testing was not an option. Since many of the findings of the current study are in line with previous laboratory-based studies in the literature, the current remote approach provides promise for future research studies using similar online techniques to collect large datasets, enhancing statistical power to detect hypothesized effects.

We must acknowledge several limitations of the current study. First, since the noise exposure questionnaire heavily relies on participants’ ability to recall the details of past noise exposure throughout their lifespan, it is possible that participants have either under- or over-estimated their lifetime noise exposure. Second, although there may be wide variability in the lifetime noise exposure scores across participants, it is hard to ascertain whether participants with the highest noise exposure scores had sufficiently high cumulative lifetime noise exposure to produce measurable effects using the different outcome measures employed.

Third, although we tried to rule out participants with a diagnosis of hearing impairment and those with a documented history of head/neck traumas, otologic pathology, ear surgeries, and ototoxic exposure, it is likely that some participants may have had a pre-existing undiagnosed age-related hearing impairment which could have influenced the findings of the current study.

Fourth, since participants used their own headphones/earphones to conduct the online SPiN task, the different headphone/earphone brands might have produced variable sound quality/level, which could add further inter-subject variability to SPiN outcomes (though DIN stimuli were low-pass filtered below 8 kHz to exclude potential influence from the high-frequency region, where the greatest variability in transducer performance is likely to be observed). Fifth, although we instructed our participants to perform the SPiN tasks in a quiet place with minimal distractions, it is possible that some participants performed the SPiN tasks in sub-optimal acoustic conditions.

Finally, the older group was smaller than the young group. This resulted in a reduced statistical power to detect the hypothesized effects of lifetime noise exposure in the older group.

## Conclusion

The findings of our study, which was carried out using novel online instruments, support the existing evidence that aging is associated with worse SPiN ability and other hearing-related symptoms such as tinnitus. However, the effect of noise exposure on tinnitus and hyperacusis was not consistent across the young and older groups. For the young group only, lifetime noise exposure was associated with a higher proportion of participants reporting tinnitus and worse severity of hyperacusis. No significant effect of lifetime noise exposure on SPiN ability, self-reported hearing, nor the severity of tinnitus handicap was found in either age group. It is not clear whether the effects of noise on the peripheral auditory system are limited, or lead to limited effects on perception, or whether the currently employed self-report and behavioral SPiN tools lack the sensitivity to detect these effects. Despite the potential lack of sensitivity, online studies are more convenient and easier to recruit participants than traditional lab-based studies and may be useful in future research efforts.

## Data Availability Statement

The datasets presented in this study can be found in online repositories. The names of the repository/repositories and accession number(s) can be found below: https://osf.io/jzu4t/files/.

## Ethics Statement

The studies involving human participants were reviewed and approved by the University of Manchester Research Ethics Committee. The patients/participants provided their written informed consent to participate in this study.

## Author Contributions

All authors listed have made a substantial, direct, and intellectual contribution to the work, and approved it for publication.

## Conflict of Interest

The authors declare that the research was conducted in the absence of any commercial or financial relationships that could be construed as a potential conflict of interest.

## Publisher’s Note

All claims expressed in this article are solely those of the authors and do not necessarily represent those of their affiliated organizations, or those of the publisher, the editors and the reviewers. Any product that may be evaluated in this article, or claim that may be made by its manufacturer, is not guaranteed or endorsed by the publisher.

## Footnotes

1
https://osf.io/jzu4t/

## Abbreviations

ARHL: Age-related hearing loss
ANFs: Auditory nerve fibers
CS: Cochlear synaptopathy
SPiN: Speech perception in noise
DIN: Digits in noise
CRM: Coordinate Response Measure
THI: Tinnitus Handicap Inventory
SSQ12: The abbreviated form of the Speech Spatial and Qualities of Hearing Questionnaire.
