# Corrigendum: The effect of lifetime noise exposure and aging on speech-perception-in-noise ability and self-reported hearing symptoms: an online study

**DOI:** 10.3389/fnagi.2023.1275798

**Published:** 2023-08-28

**Authors:** Adnan M. Shehabi, Garreth Prendergast, Hannah Guest, Christopher J. Plack

**Affiliations:** ^1^Manchester Centre for Audiology and Deafness, University of Manchester, Manchester, United Kingdom; ^2^Department of Audiology and Speech Therapy, Birzeit University, Birzeit, Palestine; ^3^Department of Psychology, Lancaster University, Lancaster, United Kingdom

**Keywords:** noise exposure, aging, cochlear synaptopathy (CS), age-related hearing loss (ARHL), speech perception in noise (SPiN), self-reported hearing, tinnitus, hyperacusis

In the published article, there was an error in the **Abstract**.

This sentence previously stated:

“Lifetime noise exposure did not predict SPiN thresholds, self-reported hearing ability, or the presence of tinnitus in either age group.”

The corrected sentence appears below:

“Lifetime noise exposure did not predict SPiN thresholds, self-reported hearing ability, or tinnitus handicap in either age group.”

We determined that our choice of statistical values to report in the **Results** section—although meeting the standards of the Editor and Reviewers—was not sufficiently informative. All statistical calculations were performed correctly, but for all linear regression models, we reported the F statistic for the entire model when referring to individual effects, when we should have reported the individual statistics. This is now provided in further detail.

In the published article there was an error to **Results**, “*Speech Perception in Noise*,” “*The Effect of Lifetime Noise Exposure*.” It previously stated:

“The primary linear regression model showed that the DIN thresholds in the older group did not vary significantly as a function of lifetime noise exposure [*R*^2^ = 0.064, *F*_(4,42)_ = 0.71, *p* = 0.337]. The covariates of sex and cognitive ability (as reflected by the forward and backward digit span scores) were not significant predictors of the DIN thresholds in the older group. The exploratory multiple linear regression models showed that lifetime noise exposure did not predict the DIN thresholds in the young group [*R*^2^ = 0.077, *F*_(4,89)_ = 1.85, *p* = 0.508]; the CRM thresholds in the older group [*R*^2^ = 0.072, *F*_(4,42)_ = 0.815, *p* = 0.852]; nor the CRM thresholds in the young group [*R*^2^ = 0.142, *F*_(4,89)_ = 3.68, *p* = 0.237].”

The corrected sentences appear below:

The primary linear regression model showed that the DIN thresholds in the older group did not vary significantly as a function of lifetime noise exposure [β = −0.14, *t* = −0.97, *p* = 0.337]. The covariates of sex and cognitive ability (as reflected by the forward and backward digit span scores) were not significant predictors of the DIN thresholds in the older group. The exploratory multiple linear regression models showed that lifetime noise exposure did not predict the DIN thresholds in the young group [β = 0.05, *t* = 0.66 *p* = 0.508]; the CRM thresholds in the older group [β = 0.12, *t* = 0.19, *p* = 0.852]; nor the CRM thresholds in the young group [β = −0.04, *t* = −1.10, *p* = 0.237].”

In the published article there was an error to **Results**, “*Speech Perception in Noise*,” “*The Effect of Age*,” It previously stated:

“The primary linear regression model showed that the DIN thresholds were significantly higher among the low-noise older participants (mean = −10.58 dB, *SD* = 1.34 dB) compared to their young low-noise counterparts [mean = −11.26 dB, *SD* = 1.15 dB; *R*^2^ = 0.083, *F*_(4,108)_ = 2.45, *p* = 0.006]. The exploratory regression model for the effect of age on CRM threshold showed that low-noise older participants performed significantly worse (i.e., higher thresholds; mean = −5.15 dB, *SD* = 5.81 dB) than their young low-noise counterparts [mean = −9.63 dB, *SD* = 5.64 dB; *R*^2^ = 0.169, *F*_(4,108)_ = 5.49, *p* = 0.001].”

The corrected sentences appear below:

“The primary linear regression model showed that the DIN thresholds were significantly higher among the low-noise older participants (mean = −10.58 dB, *SD* = 1.34 dB) compared to their young low-noise counterparts [mean = −11.26 dB, *SD* = 1.15 dB; β = 0.71, *t* = 2.83, *p* = 0.006]. The exploratory regression model for the effect of age on CRM threshold showed that low-noise older participants performed significantly worse (i.e., higher thresholds; mean = −5.15 dB, *SD* = 5.81 dB) than their young low-noise counterparts [mean = −9.63 dB, *SD* = 5.64 dB; β = 3.97, *t* = 3.40, *p* = 0.001].”

In the published article there was an error to **Results**, “*Self-Reported Hearing Ability*,” “*The Effect of Lifetime Noise Exposure*.” It previously stated:

“For the primary linear regression model for the older group, the SSQ12 scores did not vary significantly as a function of lifetime noise exposure [*R*^2^ = 0.059, *F*_(4,72)_ = 1.12, *p* = 0.06]. Neither sex nor cognitive function were significant predictors. For the young group, the exploratory regression model showed that lifetime noise exposure did not predict the SSQ12 scores [*R*^2^ = 0.04, *F*_(4,212)_ = 2.21, *p* = 0.104]. Neither sex nor cognitive function were significant predictors.”

The corrected sentences appear below:

“For the primary linear regression model for the older group, the SSQ12 scores did not vary significantly as a function of lifetime noise exposure [β = −0.24, *t* = −1.91, *p* = 0.06]. Neither sex nor cognitive function were significant predictors. For the young group, the exploratory regression model showed that lifetime noise exposure did not predict the SSQ12 scores [β = −0.09, *t* = −1.64, *p* = 0.104]. Neither sex nor cognitive function were significant predictors.”

In the published article there was an error to **Results**, “*Self-Reported Hearing Ability*,” “*The Effect of Age*.” It previously stated:

“As per the primary linear regression model, older low-noise participants had similar SSQ12 scores (mean = 7.43, *SD* = 1.64 dB) compared to their low-noise young counterparts (mean = 7.72, *SD* = 1.32) [*R*^2^ = 0.03, *F*_(4,225)_ = 1.76, *p* = 0.787].”

The corrected sentences appear below:

“As per the primary linear regression model, older low-noise participants had similar SSQ12 scores (mean = 7.43, *SD* = 1.64 dB) compared to their low-noise young counterparts (mean = 7.72, *SD* = 1.32) [β = −0.27, *t* = −1.25, *p* = 0.211].”

In the published article there was an error to **Results**, “*Tinnitus*,” “*The Effect of Lifetime Noise Exposure*.” It previously stated:

“Exploratory linear regression models showed that lifetime noise exposure did not predict the THI scores in the young [*R*^2^ = 0.05, *F*_(4,35)_ = 0.49, *p* = 0.307] nor older [*R*^2^ = 0.09, *F*_(4,21)_ = 0.49, *p* = 0.461] groups. Neither sex nor cognitive function were significant predictors.”

The corrected sentences appear below:

“Exploratory linear regression models showed that lifetime noise exposure did not predict the THI scores in the young [β = 1.47, *t* = 1.04, *p* = 0.307] nor older [β = 1.56, *t* = 0.75, *p* = 0.461] groups. Neither sex nor cognitive function were significant predictors.”

In the published article there was an error to **Results**, “*Tinnitus*,” “*The Effect of Age*.” It previously stated:

“The exploratory regression models showed that the THI scores were similar across low-noise participants in the young (mean = 10.57, *SD* = 8.76) and older (mean = 12.44, *SD* = 10.55) groups [*R*^2^ = 0.04, *F*_(4,44)_ = 0.40, *p* = 0.448]. Neither sex nor cognitive function were significant predictors.”

The corrected sentences appear below:

“The exploratory regression models showed that the THI scores were similar across low-noise participants in the young (mean = 10.57, *SD* = 8.76) and older (mean = 12.44, *SD* = 10.55) groups [β = 2.34, *t* = 0.77, *p* = 0.448]. Neither sex nor cognitive function were significant predictors.”

In the published article there was an error to **Results**, “*Hyperacusis*,” “*The Effect of Lifetime Noise Exposure*.” It previously stated:

“The exploratory linear regression models showed that hyperacusis scores in the young group were significantly higher as lifetime noise exposure increased [*R*^2^ = 0.05, *F*_(4, 212)_ = 2.81, *p* = 0.001]. For the older group, lifetime noise exposure did not predict hyperacusis scores [*R*^2^ = 0.049, *F*_(4,72)_ = 0.93, *p* = 0.812]. Neither sex nor cognitive function were significant predictors in either model.”

The corrected sentences appear below:

“The exploratory linear regression models showed that hyperacusis scores in the young group were significantly higher as lifetime noise exposure increased [β = 0.07, *t* = 3.27, *p* = 0.001]. For the older group, lifetime noise exposure did not predict hyperacusis scores [β = 0.01, *t* = 0.24, *p* = 0.812]. Neither sex nor cognitive function were significant predictors in either model.”

In the published article there was an error to **Results**, “*Hyperacusis*,” “*The Effect of Age*.” It previously stated:

“The exploratory linear regression model showed that the hyperacusis scores of low-noise young participants (mean = 0.78, *SD* = 0.50) did not differ significantly from those of their older counterparts [mean = 0.82, *SD* = 0.55; *R*^2^ = 0.006, *F*_(4,225)_ = 0.33, *p* = 0.611]. Neither sex nor cognitive function were significant predictors.”

The corrected sentences appear below:

“The exploratory linear regression model showed that the hyperacusis scores of low-noise young participants (mean = 0.78, *SD* = 0.50) did not differ significantly from those of their older counterparts [mean = 0.82, *SD* = 0.55; β = 0.041, *t* = 0.510, *p* = 0.611]. Neither sex nor cognitive function were significant predictors.”

The authors apologize for this error and state that this does not change the scientific conclusions of the article in any way. The original article has been updated.

