# Supplementary material for: The Effect of Lifetime Noise Exposure and Aging on Speech-Perception-in-Noise Ability and Self-Reported Hearing Symptoms: An Online Study
Source: Front Aging Neurosci. 2022 May 30;14:890010. doi: 10.3389/fnagi.2022.890010 (PMC9195834; doi:10.3389/fnagi.2022.890010)
Supplement: Supplementary file 1 [file Data_Sheet_1.pdf]

AD8 Dementia Screening Tool

Please complete the AD8 Dementia Screening Questionnaire

Thank you!

In this section, we will ask you about some situations related to your memory abilities over the past months and years. Please judge whether you think there has been a change to each memory ability by checking the appropriate box next to each statement.

Please Remember: "Yes, a change" indicates that there has been a change in this ability over the last several months or years. If a statement does not apply to you, please select the N/A (Not applicable) button.

	YES, a change	NO, No change	N/A
1. Problems with judgment (e.g., problems making decisions, bad financial decisions, problems with thinking)	○	○	○
2. Less interest in hobbies/activities	○	○	○
3. Repeats the same things over and over (questions, stories, or statements)	○	○	○
4. Trouble learning how to use a tool, appliance, or gadget (e.g., VCR, computer, microwave, remote control)	○	○	○
5. Forget correct month or year	○	○	○
6. Trouble handling complicated financial affairs (e.g., balancing checkbook, income taxes, paying bills)	○	○	○
7. Trouble remembering appointments	○	○	○
8. Daily problems with thinking and/or memory	○	○	○

Clinical And Demographic Questionnaire

Please complete the Clinical and Demographic Questionnaire below

Thank you!

In this section, we will ask you questions related to your demographic information, hearing health and general medical status.

Please enter your email address that you would like us to contact you on if needed:<br><br>Note: we may contact you regarding GP referral, prize draw outcome or to invite you to participate in future research (if you have already given consent to this)	________________________
OPTIONAL:<br>Please enter your phone number that you would like us to contact you on if needed	________________________
Please select your gender	◯ Male<br>◯ Female<br>◯ Other
Please specify (optional)	________________________
Please enter your age as at your last birthday	________________________<br>(E.g. 55 years)
Have you been exposed to loud sounds over the past 24 hours (e.g. sounds that are as loud as nightclub music)	◯ Yes<br>◯ No
Please specify the type(s) of loud noise that you have been exposed to in the past 24 hours?	________________________
Please specify how many hours and minutes (approximately) you have been exposed to loud noises in the past 24 hours?	________________________<br>(E.g. 3 hours 30 minutes)
Do you consider yourself to have a current hearing impairment?	◯ Yes<br>◯ No
In which ear do you think you have hearing impairment?	◯ Left<br>◯ Right<br>◯ Both<br>◯ Not sure
Do you think your hearing sensitivity changes from day-to-day?	◯ No<br>◯ Yes (associated with dizziness)<br>◯ Yes (not associated with dizziness)
Please give more detail regarding the day-to-day changes in your hearing sensitivity? (optional)	______________________________

Do you think you currently have chronic dizziness?	◯ Yes<br>◯ No
Regarding your dizziness, please specify:<br><br>On topline: when the chronic dizziness started (month/year).<br><br>On second line: Whether the dizziness is associated with a diagnosed medical condition	______________________________
Do you currently suffer from tinnitus?<br><br>Tinnitus involves hearing sounds when it's quiet or a persistent ringing, buzzing or other types of noise without an external source	◯ Yes<br>◯ No
Do you have any specific medical conditions / disabilities?	◯ Yes<br>◯ No
Please list any medical conditions you may have?	______________________________
Do you smoke?	◯ No<br>◯ Occasionally<br>◯ Less than 10 cigarettes a day<br>◯ 10-20 cigarettes a day<br>◯ More than 20 cigarettes a day
Please specify how many years you have been smoking approximately?	________________________<br>(E.g. 8 years)
Other than hearing loss due to old age, do you have any family history of hearing impairment?	◯ Yes<br>◯ No<br>◯ I don't know<br>◯ Rather not say
Have you recently had an infection of the middle ear (glue ear)?	◯ No<br>◯ Yes (controlled with antibiotics)<br>◯ Yes (requiring a grommet)<br>◯ Yes (resolved spontaneously)<br>◯ I don't know
Which ear you did you have the infection in?	◯ Left Ear<br>◯ Right Ear<br>◯ Both ears
Have you ever had ear surgery?	◯ No<br>◯ Yes - right ear<br>◯ Yes - left ear<br>◯ Yes - both ears

Regarding your ear surgery(ies), please specify:

On topline: when (year) did you have the ear surgery? ______________________________

Second line: The reason for surgery

Have you ever taken any of the following medications? (Please tick all choices that apply)

- ☐ Non-Steroidal Anti-Inflammatory Drugs (NSAIDs), including aspirin, ibuprofen, and naproxen
- ☐ Water pills and diuretics
- ☐ Chemotherapy drugs including cisplatin
- ☐ Allergy medications
- ☐ Antimalarial medications
- ☐ Tricyclic antidepressants
- ☐ Anti-anxiety medications
- ☐ Antibiotics
- ☐ None of the above

For each of the selected medications from the above list, Please specify:

1- On topline: How many months and years approximately you have taken EACH medication

2- On Second line: The daily/weekly dosage of EACH medication

____

Do you consume alcohol?

- ◯ No
- ◯ Yes, monthly or less
- ◯ Yes, 2-4 times a month
- ◯ Yes, 2-3 times a week
- ◯ Yes, 4 or more times a week

Do you think you have any memory problems?

- ◯ Yes
- ◯ No

Please explain the nature of these memory problems (Optional)

____

Have you worked in a noisy workplace/job for at least 5 years?

- ◯ Yes
- ◯ No

Did you regularly wear hearing protection (e.g. earplugs/ear defenders) while working in this noisy workplace?

- ◯ Yes
- ◯ No

Please specify the type of workplace/job where you were exposed to loud noises

____
(E.g. Construction work)

What is your highest level of education / training?

- ◯ Primary school
- ◯ GCSEs / GCE O-Levels / SAT I
- ◯ GCE A-Levels / SAT II / High School Certificate
- ◯ Apprenticeship / Vocational training
- ◯ Undergraduate university degree (e.g. BA/BSc)
- ◯ Postgraduate university degree (e.g. MA/ MSc / PhD)

Is English your first language

- ◯ Yes
- ◯ No

What is your first language?

____

Hyperacusis Questionnaire

Please complete Hyperacusis Questionnaire

Thank you!

PURPOSE: This section contains 14 questions on your sensitivity to sound and other noises in your environment.

For each question, please select the answer choice which best applies to you. By selecting (No), it means that the statement is never true for you. By selecting (Yes, a lot), it means that the statement is almost always true for you.

	No	Yes, a little	Yes, quite a lot	Yes, a lot
1. Do you ever use earplugs or earmuffs to reduce your noise perception? (Do not consider the use of hearing protection during abnormally high noise exposure situations.)	○	○	○	○
2. Do you find it harder to ignore sound around you in everyday situations?	○	○	○	○
3. Do you have trouble reading in a noisy or loud environment?	○	○	○	○
4. Do you have trouble concentrating in a noisy or loud environment?	○	○	○	○
5. Do you have difficulty listening to conversations in noisy places?	○	○	○	○
6. Has anyone you know ever told you that you tolerate noise or certain kinds of sound badly?	○	○	○	○
7. Are you particularly sensitive to or bothered by street noise?	○	○	○	○
8. Do you find the noise unpleasant in certain social situations (e.g., night clubs, pubs or bars, concerts, firework displays, cocktail receptions)?	○	○	○	○

9. When someone suggests doing something (going out, to the cinema, to a concert, etc.), do you immediately think about the noise you are going to have to put up with?	○	○	○	○
10. Do you ever turn down an invitation or not go out because of the noise you would have to face?	○	○	○	○
11. Do noises or particular sounds bother you more in a quiet place than in a slightly noisy room?	○	○	○	○
12. Do stress and tiredness reduce your ability to concentrate in noise?	○	○	○	○
13. Are you less able to concentrate in noise toward the end of the day?	○	○	○	○
14. Do noise and certain sounds cause you stress and irritation?	○	○	○	○

Noise Exposure Questionnaire

Please complete the Noise Exposure Questionnaire

Thank you!

The purpose of this questionnaire is to estimate your lifetime noise exposure. We'll ask you to identify activities that have caused you to be in noisy situations, then estimate how long you spent in those situations and how noisy they were.

The questionnaire is divided into four sections:
Section A: Occupational and education-related noise exposure
Section B: Recreational noise exposure
Section C: Earphone and headphone noise exposure
Section D: Firearm noise exposure

Please answer all the questions in all sections, either by ticking boxes or typing in the requested information.

SECTION A: OCCUPATIONAL AND EDUCATIONAL NOISE EXPOSURE

In this section, we'll ask you to tell us about any noisy situations you've been exposed to while at work or in education.

By "noisy", we mean situations where you would have to raise your voice to communicate (with a person standing 4 feet away who has normal hearing and isn't wearing hearing protection).

In your lifetime, how many different noisy jobs (or educational situations) have you had?

○ 0 ○ 1 ○ 2 ○ 3
○ 4 ○ 5

Now tell us about the FIRST of these noisy occupational/educational situations

What was the noisy job/situation?

____

Was it loud enough that you would have needed to raise your voice to communicate with someone 4 feet away?

◯ Yes
◯ No

Tell us approximately how long you were exposed, by writing the following four numbers on separate lines.

Top line: How many years were you exposed?
Second line: How many weeks per year (on average)?
Third line: How many days per week (on average)?
Fourth line: How many hours per day (on average)?

____

Tell us how loud it was, on average:

If you were trying to communicate with someone 4 feet away (with normal hearing and without hearing protection), how loud would you have to speak?

◯ Talk normally from 4 feet
◯ Raise voice from 4 feet
◯ Talk loudly from 4 feet
◯ Talk very loudly from 4 feet
◯ Shout from 4 feet
◯ Shout from 2 feet
◯ Shout in ear

Did you ever wear hearing protection (e.g. earplugs or earmuffs)?

◯ Yes
◯ No

What percentage of the time did you wear hearing protection?

◯ 0% ◯ 10% ◯ 20% ◯ 30% ◯ 40% ◯ 50% ◯ 60% ◯ 70% ◯ 80% ◯ 90%
◯ 100%

These are different types of hearing protection numbered from 1 to 7

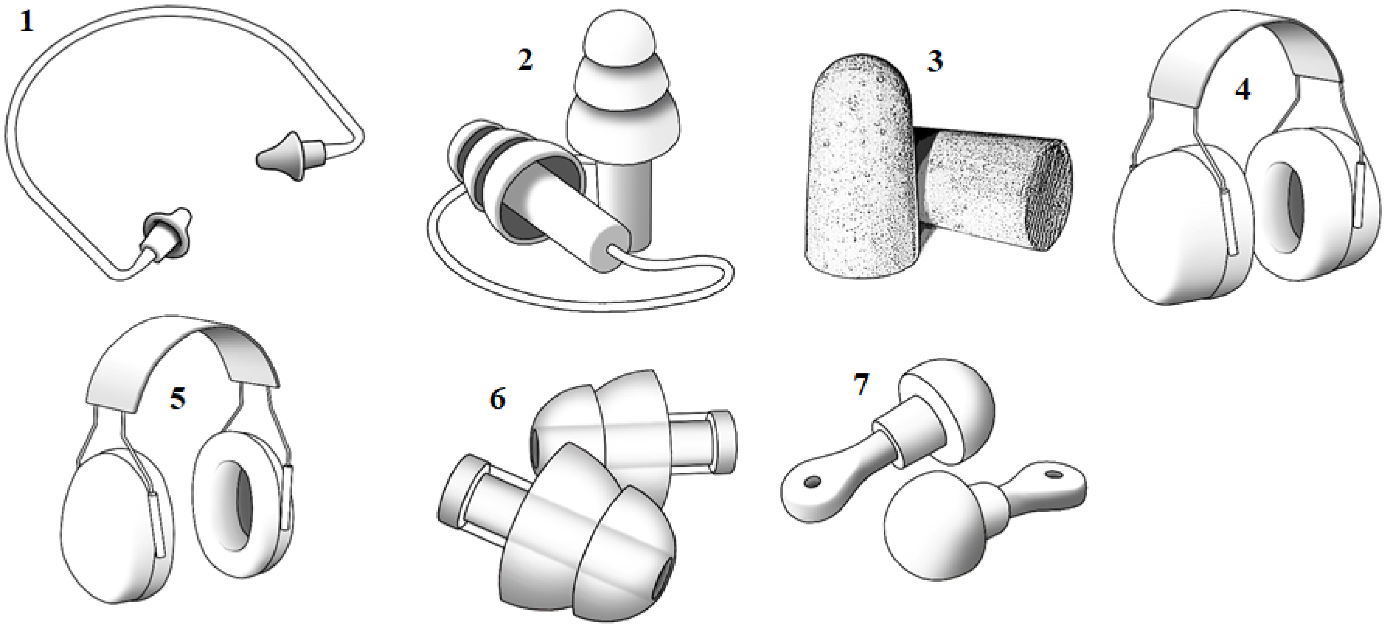

Select the kind of hearing protection you wore

☐ 1 ☐ 2 ☐ 3 ☐ 4
☐ 5 ☐ 6 ☐ 7

Now tell us about the SECOND of these noisy occupational/educational situations

What was the noisy job/situation?

____

Was it loud enough that you would have needed to raise your voice to communicate with someone 4 feet away?

◯ Yes
◯ No

Tell us approximately how long you were exposed, by writing the following four numbers on separate lines.

Top line: How many years were you exposed?
Second line: How many weeks per year (on average)?
Third line: How many days per week (on average)?
Fourth line: How many hours per day (on average)?

____

Tell us how loud it was, on average:

If you were trying to communicate with someone 4 feet away (with normal hearing and without hearing protection), how loud would you have to speak?

◯ Talk normally from 4 feet
◯ Raise voice from 4 feet
◯ Talk loudly from 4 feet
◯ Talk very loudly from 4 feet
◯ Shout from 4 feet
◯ Shout from 2 feet
◯ Shout in ear

Did you ever wear hearing protection (e.g. earplugs or earmuffs)?

◯ Yes
◯ No

What percentage of the time did you wear hearing protection?

◯ 0% ◯ 10% ◯ 20% ◯ 30% ◯ 40% ◯ 50% ◯ 60% ◯ 70% ◯ 80% ◯ 90%
◯ 100%

These are different types of hearing protection numbered from 1 to 7

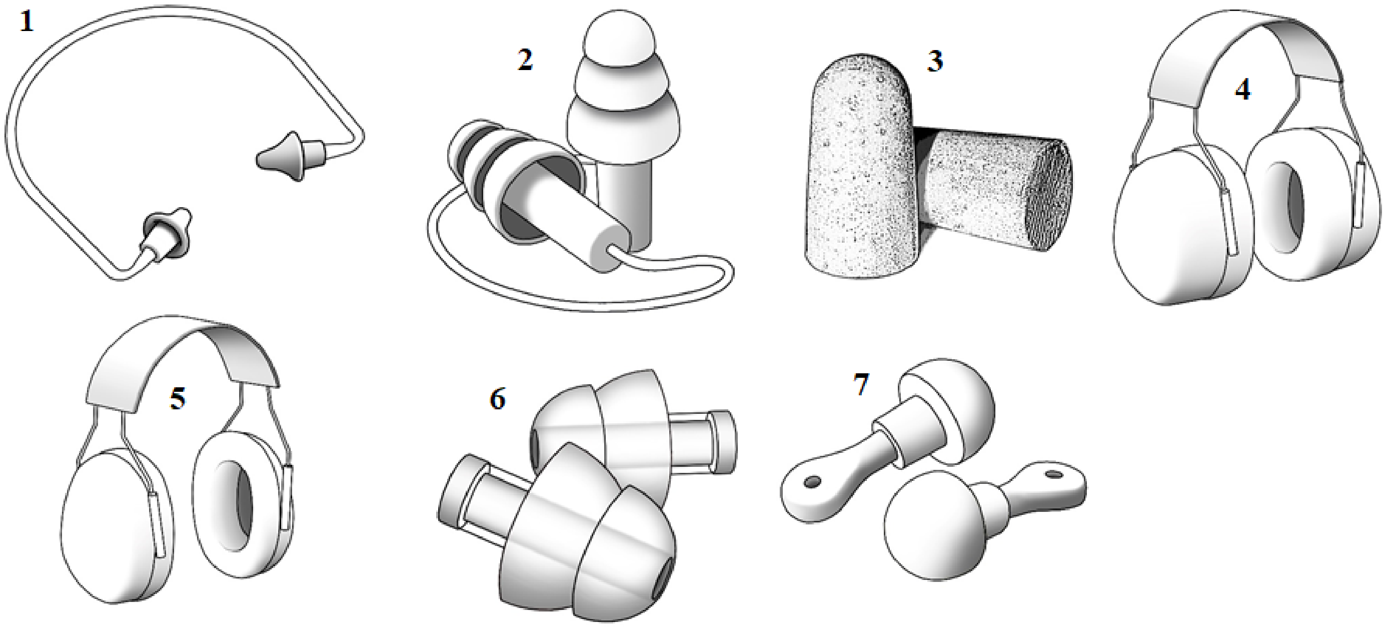

Select the kind of hearing protection you wore

☐ 1 ☐ 2 ☐ 3 ☐ 4
☐ 5 ☐ 6 ☐ 7

Now tell us about the THIRD of these noisy occupational/educational situations

What was the noisy job/situation?

____

Was it loud enough that you would have needed to raise your voice to communicate with someone 4 feet away?

◯ Yes
◯ No

Tell us approximately how long you were exposed, by writing the following four numbers on separate lines.

Top line: How many years were you exposed?
Second line: How many weeks per year (on average)?
Third line: How many days per week (on average)?
Fourth line: How many hours per day (on average)?

____

Tell us how loud it was, on average:

If you were trying to communicate with someone 4 feet away (with normal hearing and without hearing protection), how loud would you have to speak?

◯ Talk normally from 4 feet
◯ Raise voice from 4 feet
◯ Talk loudly from 4 feet
◯ Talk very loudly from 4 feet
◯ Shout from 4 feet
◯ Shout from 2 feet
◯ Shout in ear

Did you ever wear hearing protection (e.g. earplugs or earmuffs)?

◯ Yes
◯ No

What percentage of the time did you wear hearing protection?

◯ 0% ◯ 10% ◯ 20% ◯ 30% ◯ 40% ◯ 50% ◯ 60% ◯ 70% ◯ 80% ◯ 90%
◯ 100%

These are different types of hearing protection numbered from 1 to 7

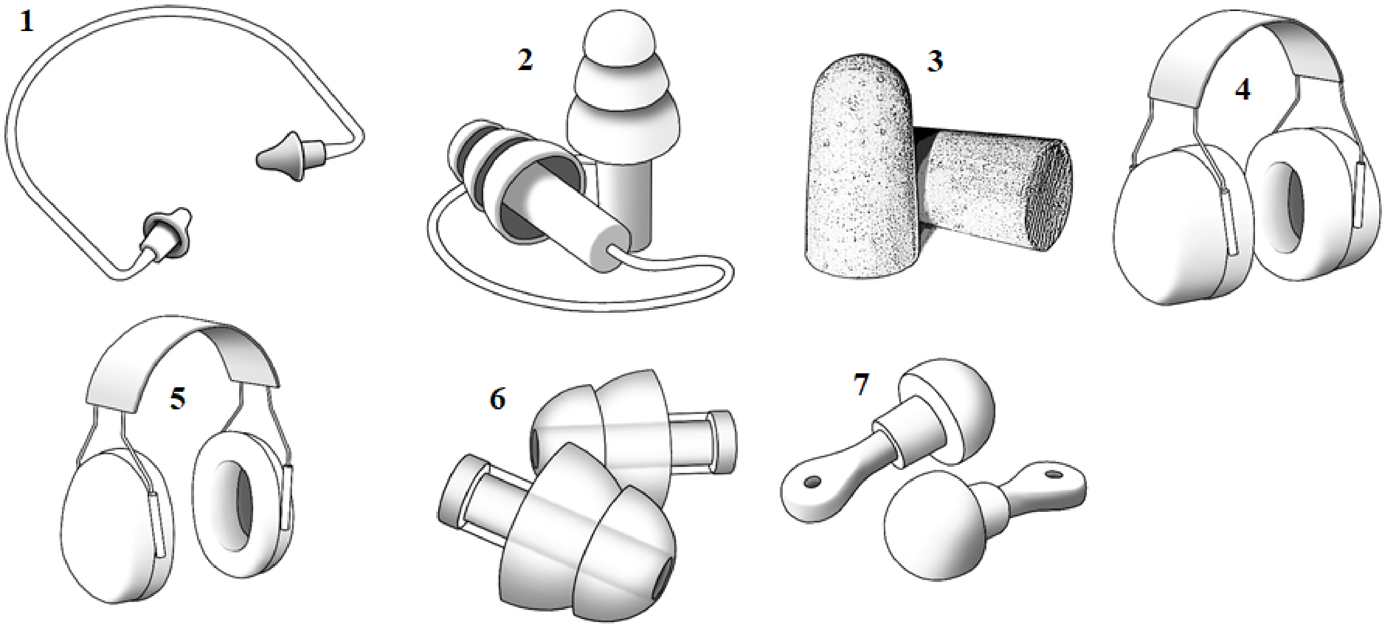

Select the kind of hearing protection you wore

☐ 1 ☐ 2 ☐ 3 ☐ 4
☐ 5 ☐ 6 ☐ 7

Now tell us about the FOURTH of these noisy occupational/educational situations

What was the noisy job/situation? ______________________________

Was it loud enough that you would have needed to raise your voice to communicate with someone 4 feet away?
- ◯ Yes
- ◯ No

Tell us approximately how long you were exposed, by writing the following four numbers on separate lines. ______________________________________

Top line: How many years were you exposed?
Second line: How many weeks per year (on average)?
Third line: How many days per week (on average)?
Fourth line: How many hours per day (on average)?

Tell us how loud it was, on average:

If you were trying to communicate with someone 4 feet away (with normal hearing and without hearing protection), how loud would you have to speak?
- ◯ Talk normally from 4 feet
- ◯ Raise voice from 4 feet
- ◯ Talk loudly from 4 feet
- ◯ Talk very loudly from 4 feet
- ◯ Shout from 4 feet
- ◯ Shout from 2 feet
- ◯ Shout in ear

Did you ever wear hearing protection (e.g. earplugs or earmuffs)?
- ◯ Yes
- ◯ No

What percentage of the time did you wear hearing protection?

◯ 0% ◯ 10% ◯ 20% ◯ 30% ◯ 40% ◯ 50% ◯ 60% ◯ 70% ◯ 80% ◯ 90% ◯ 100%

These are different types of hearing protection numbered from 1 to 7

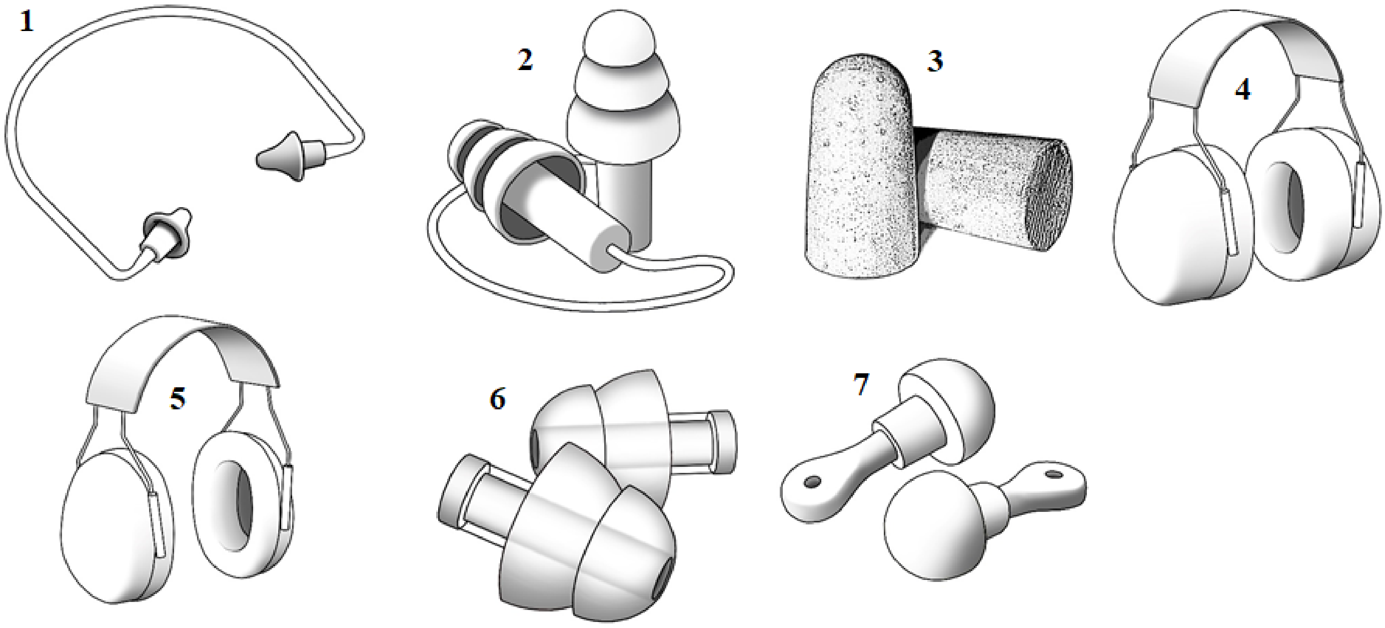

Select the kind of hearing protection you wore

☐ 1 ☐ 2 ☐ 3 ☐ 4
☐ 5 ☐ 6 ☐ 7

Now tell us about the FIFTH of these noisy occupational/educational situations

What was the noisy job/situation?

____

Was it loud enough that you would have needed to raise your voice to communicate with someone 4 feet away?

◯ Yes
◯ No

Tell us approximately how long you were exposed, by writing the following four numbers on separate lines.

Top line: How many years were you exposed?
Second line: How many weeks per year (on average)?
Third line: How many days per week (on average)?
Fourth line: How many hours per day (on average)?

____

Tell us how loud it was, on average:

If you were trying to communicate with someone 4 feet away (with normal hearing and without hearing protection), how loud would you have to speak?

◯ Talk normally from 4 feet
◯ Raise voice from 4 feet
◯ Talk loudly from 4 feet
◯ Talk very loudly from 4 feet
◯ Shout from 4 feet
◯ Shout from 2 feet
◯ Shout in ear

Did you ever wear hearing protection (e.g. earplugs or earmuffs)?

◯ Yes
◯ No

What percentage of the time did you wear hearing protection?

◯ 0% ◯ 10% ◯ 20% ◯ 30% ◯ 40% ◯ 50% ◯ 60% ◯ 70% ◯ 80% ◯ 90%
◯ 100%

These are different types of hearing protection numbered from 1 to 7

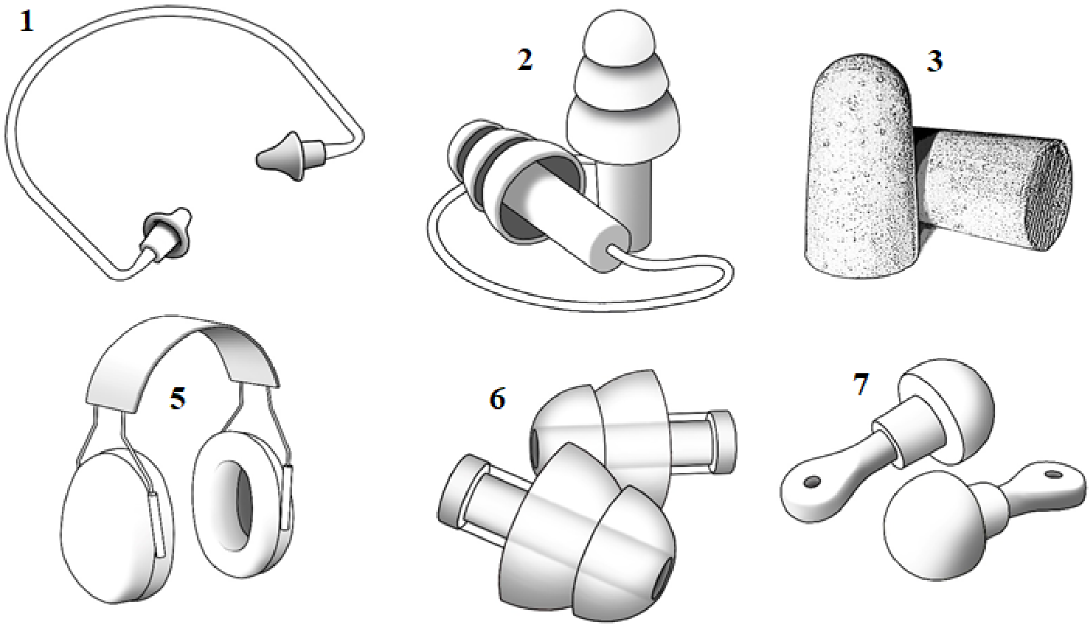

Select the kind of hearing protection you wore	☐ 1 ☐ 2 ☐ 3 ☐ 4<br>☐ 5 ☐ 6 ☐ 7

SECTION B: RECREATIONAL NOISE EXPOSURE

In this section, we'll ask you to tell us about your lifetime exposure to noisy recreational situations.

By "noisy", we mean situations where you would have to raise your voice to communicate (with a person standing 4 feet away who has normal hearing and isn't wearing hearing protection).

By "recreational", we mean any situations you've encountered that DIDN'T take place at work or in education.

B. Please tick ALL boxes which correspond to noisy recreational situations that you have been exposed to.

- ☐ Concerts with amplified music
- ☐ Music festivals
- ☐ Nightclubs and bars
- ☐ Making music (e.g. playing, DJing, singing)
- ☐ Power tools (including powered gardening tools)
- ☐ Engine noise (e.g. motorbikes, motorsports)
- ☐ Sport-related noise (e.g. sports matches)
- ☐ Cinema
- ☐ Other
- ☐ None of the above

In this subsection, we'll ask for more information on your exposure to concerts with amplified music

Were the concerts loud enough that you would have needed to raise your voice to communicate with someone 4 feet away?

◯ Yes
◯ No

Tell us approximately how long you were exposed, by writing the following four numbers on separate lines.

Top line: How many years were you exposed?
Second line: How many weeks per year (on average)?
Third line: How many days per week (on average)?
Fourth line: How many hours per day (on average)?

____

Tell us how loud the concerts were, on average:

If you were trying to communicate with someone 4 feet away (with normal hearing and without hearing protection), how loud would you have to speak?

◯ Talk normally from 4 feet
◯ Raise voice from 4 feet
◯ Talk loudly from 4 feet
◯ Talk very loudly from 4 feet
◯ Shout from 4 feet
◯ Shout from 2 feet
◯ Shout in ear

Did you ever wear hearing protection (e.g. earplugs or earmuffs) when at concerts with amplified music?

◯ Yes
◯ No

What percentage of the time did you wear the hearing protection?

◯ 0% ◯ 10% ◯ 20% ◯ 30% ◯ 40% ◯ 50% ◯ 60% ◯ 70% ◯ 80% ◯ 90% ◯ 100%

These are different types of hearing protection numbered from 1 to 7

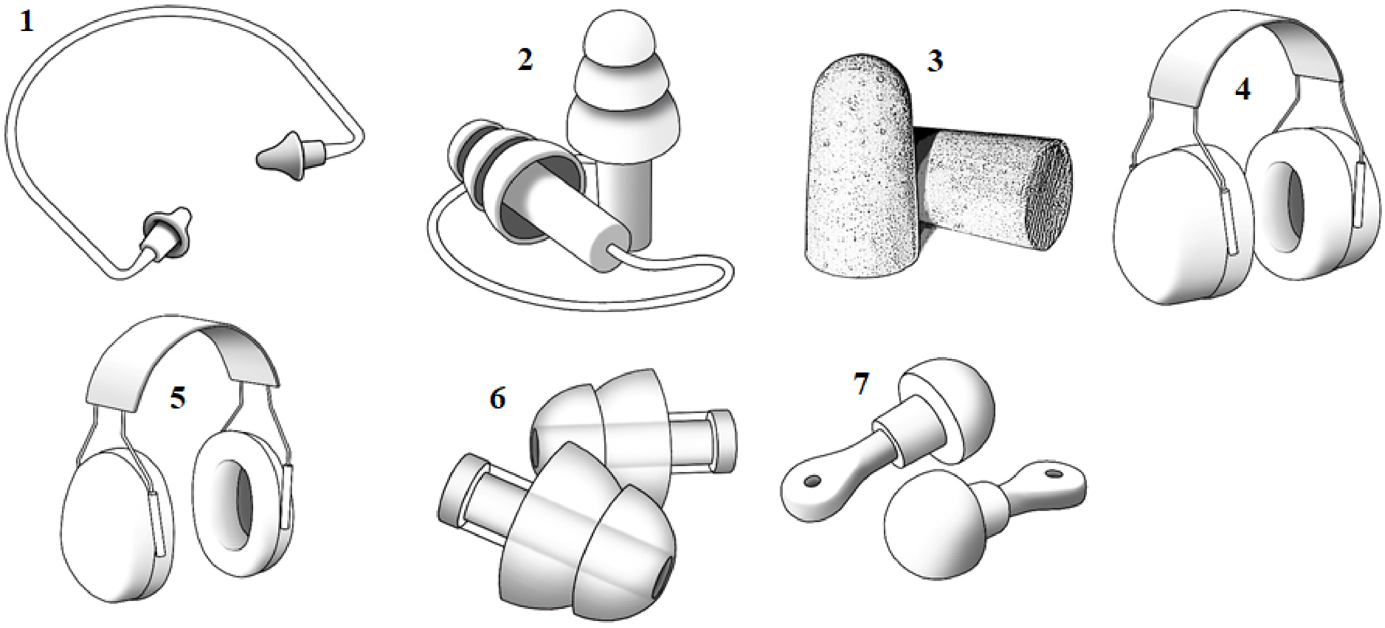

Select the kind of hearing protection you wore

☐ 1 ☐ 2 ☐ 3 ☐ 4
☐ 5 ☐ 6 ☐ 7

In this subsection, we'll ask for more information on your exposure to music festivals

Were the music festivals loud enough that you would have needed to raise your voice to communicate with someone 4 feet away?

◯ Yes
◯ No

Tell us approximately how long you were exposed, by writing the following four numbers on separate lines.

Top line: How many years were you exposed?
Second line: How many weeks per year (on average)?
Third line: How many days per week (on average)?
Fourth line: How many hours per day (on average)?

____

Tell us how loud the music festivals were, on average:

If you were trying to communicate with someone 4 feet away (with normal hearing and without hearing protection), how loud would you have to speak?

◯ Talk normally from 4 feet
◯ Raise voice from 4 feet
◯ Talk loudly from 4 feet
◯ Talk very loudly from 4 feet
◯ Shout from 4 feet
◯ Shout from 2 feet
◯ Shout in ear

Did you ever wear hearing protection (e.g. earplugs or earmuffs) when exposed to music festivals?

◯ Yes
◯ No

What percentage of the time did you wear the hearing protection?

◯ 0% ◯ 10% ◯ 20% ◯ 30% ◯ 40% ◯ 50% ◯ 60% ◯ 70% ◯ 80% ◯ 90%
◯ 100%

These are different types of hearing protection numbered from 1 to 7

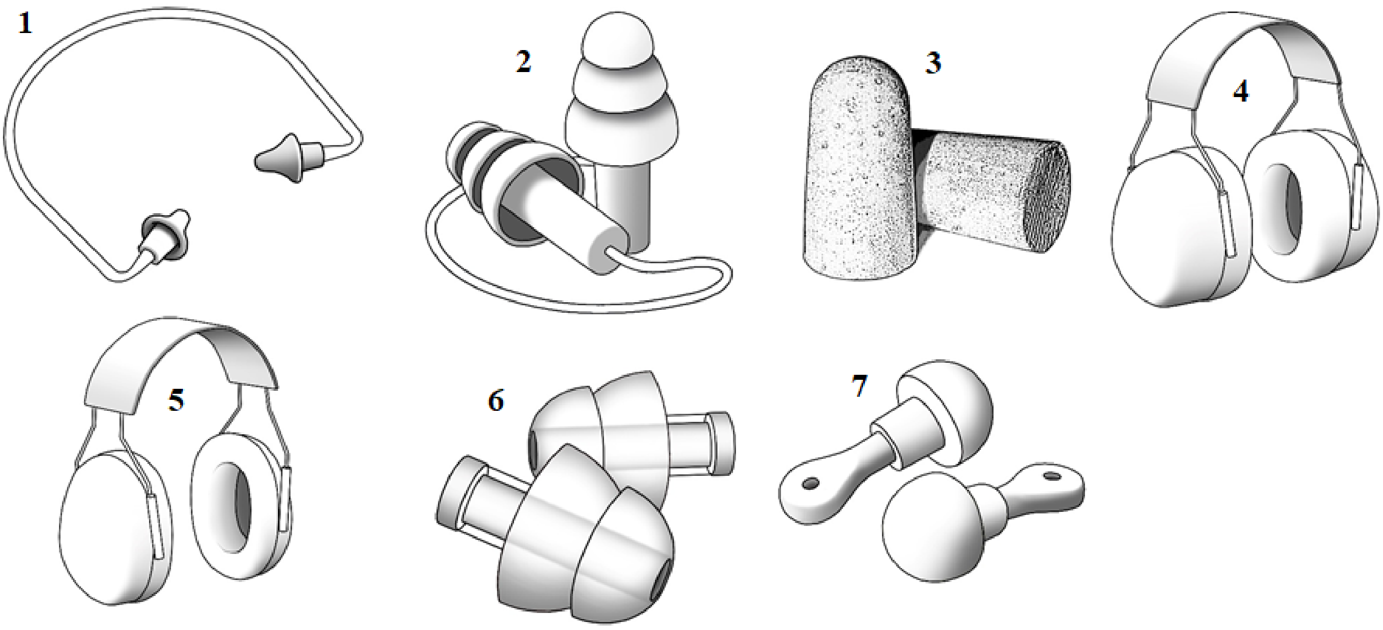

Select the kind of hearing protection you wore

☐ 1 ☐ 2 ☐ 3 ☐ 4
☐ 5 ☐ 6 ☐ 7

In this subsection, we'll ask for more information on your exposure to noise in nightclubs and bars

Were the nightclubs/bars loud enough that you would have needed to raise your voice to communicate with someone 4 feet away?

◯ Yes
◯ No

Tell us approximately how long you were exposed, by writing the following four numbers on separate lines.

Top line: How many years were you exposed?
Second line: How many weeks per year (on average)?
Third line: How many days per week (on average)?
Fourth line: How many hours per day (on average)?

____

Tell us how loud the nightclubs/bars were, on average:

If you were trying to communicate with someone 4 feet away (with normal hearing and without hearing protection), how loud would you have to speak?

◯ Talk normally from 4 feet
◯ Raise voice from 4 feet
◯ Talk loudly from 4 feet
◯ Talk very loudly from 4 feet
◯ Shout from 4 feet
◯ Shout from 2 feet
◯ Shout in ear

Did you ever wear hearing protection (e.g. earplugs or earmuffs) when exposed to music in nightclubs/bars?

◯ Yes
◯ No

What percentage of the time did you wear the hearing protection?

◯ 0% ◯ 10% ◯ 20% ◯ 30% ◯ 40% ◯ 50% ◯ 60% ◯ 70% ◯ 80% ◯ 90%
◯ 100%

These are different types of hearing protection numbered from 1 to 7

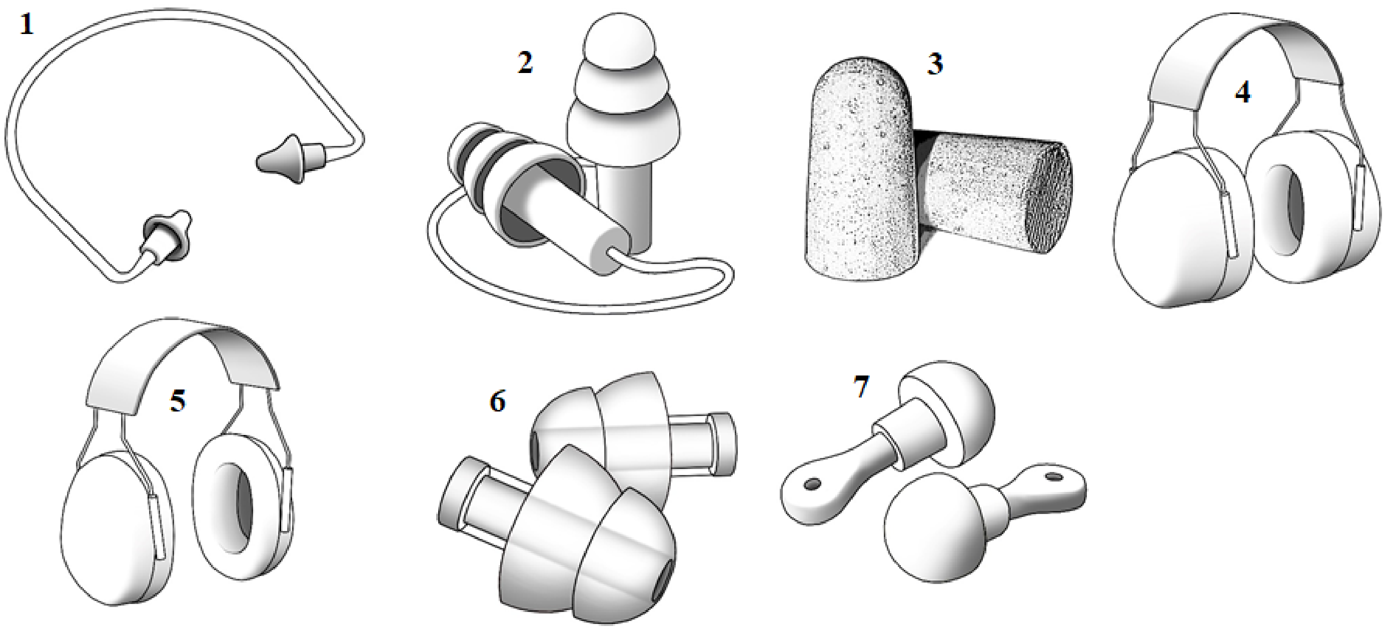

Select the kind of hearing protection you wore

☐ 1 ☐ 2 ☐ 3 ☐ 4
☐ 5 ☐ 6 ☐ 7

In this subsection, we'll ask for more information on your noise exposure while making music (playing, singing and/or DJing)

When making music, was it loud enough that you would have needed to raise your voice to communicate with someone 4 feet away?

◯ Yes
◯ No

Tell us approximately how long you were exposed, by writing the following four numbers on separate lines.

Top line: How many years were you exposed?
Second line: How many weeks per year (on average)?
Third line: How many days per week (on average)?
Fourth line: How many hours per day (on average)?

____

Tell us how loud it was when you made music, on average:

If you were trying to communicate with someone 4 feet away (with normal hearing and without hearing protection), how loud would you have to speak?

◯ Talk normally from 4 feet
◯ Raise voice from 4 feet
◯ Talk loudly from 4 feet
◯ Talk very loudly from 4 feet
◯ Shout from 4 feet
◯ Shout from 2 feet
◯ Shout in ear

Did you ever wear hearing protection (e.g. earplugs or earmuffs) when making music?

◯ Yes
◯ No

What percentage of the time did you wear the hearing protection when making music?

◯ 0% ◯ 10% ◯ 20% ◯ 30% ◯ 40% ◯ 50% ◯ 60% ◯ 70% ◯ 80% ◯ 90% ◯ 100%

These are different types of hearing protection numbered from 1 to 7

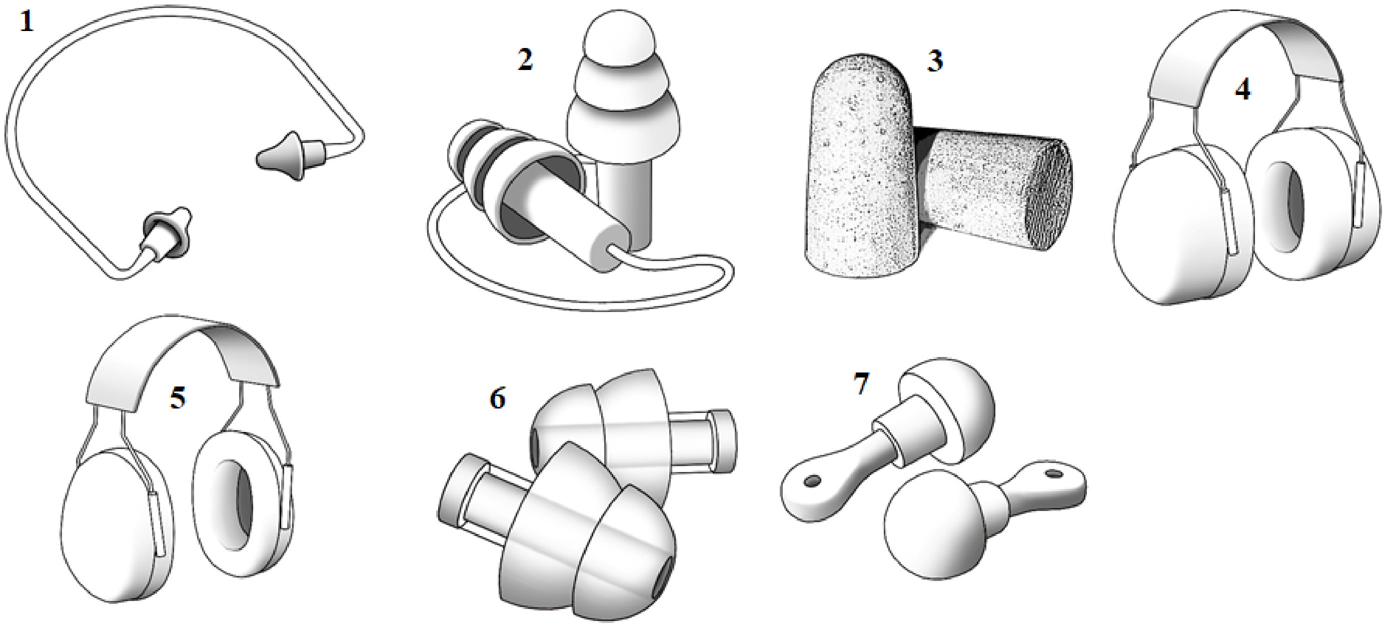

Select the kind of hearing protection you wore when making music	☐ 1 ☐ 2 ☐ 3 ☐ 4<br>☐ 5 ☐ 6 ☐ 7

In this subsection, we'll ask for more information on your exposure to noise from power tools (including powered gardening tools)

Were the power tools loud enough that you would have needed to raise your voice to communicate with someone 4 feet away?

◯ Yes
◯ No

Tell us approximately how long you were exposed, by writing the following four numbers on separate lines.

Top line: How many years were you exposed?
Second line: How many weeks per year (on average)?
Third line: How many days per week (on average)?
Fourth line: How many hours per day (on average)?

____

Tell us how loud the power tools were, on average:

If you were trying to communicate with someone 4 feet away (with normal hearing and without hearing protection), how loud would you have to speak?

◯ Talk normally from 4 feet
◯ Raise voice from 4 feet
◯ Talk loudly from 4 feet
◯ Talk very loudly from 4 feet
◯ Shout from 4 feet
◯ Shout from 2 feet
◯ Shout in ear

Did you ever wear hearing protection (e.g. earplugs or earmuffs) when exposed to noise from power tools?

◯ Yes
◯ No

What percentage of the time did you wear the hearing protection?

◯ 0% ◯ 10% ◯ 20% ◯ 30% ◯ 40% ◯ 50% ◯ 60% ◯ 70% ◯ 80% ◯ 90%
◯ 100%

These are different types of hearing protection numbered from 1 to 7

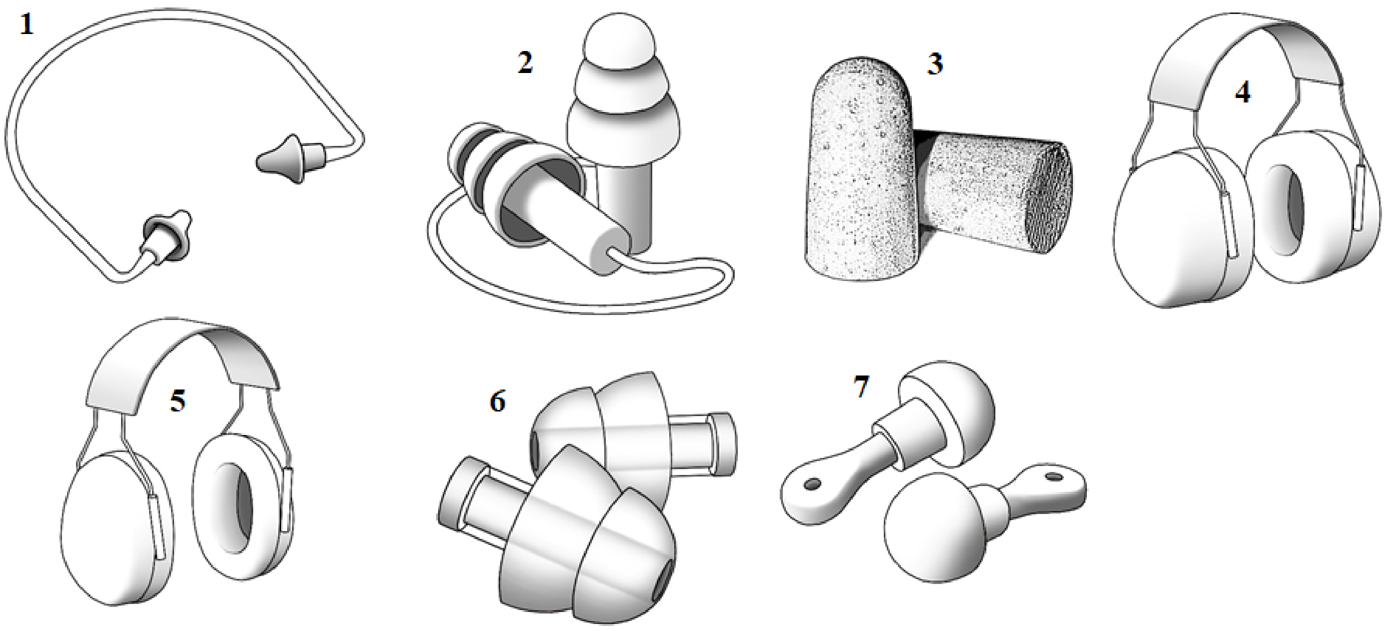

Select the kind of hearing protection you wore	☐ 1 ☐ 2 ☐ 3 ☐ 4<br>☐ 5 ☐ 6 ☐ 7

In this subsection, we'll ask for more information on your exposure to engine noises (e.g. motorbikes and motorsports)

Was the engine noise loud enough that you would have needed to raise your voice to communicate with someone 4 feet away?

◯ Yes
◯ No

Tell us approximately how long you were exposed to engine noise, by writing the following four numbers on separate lines.

Top line: How many years were you exposed?
Second line: How many weeks per year (on average)?
Third line: How many days per week (on average)?
Fourth line: How many hours per day (on average)?

____

Tell us how loud the engine noise was, on average:

If you were trying to communicate with someone 4 feet away (with normal hearing and without hearing protection), how loud would you have to speak?

◯ Talk normally from 4 feet
◯ Raise voice from 4 feet
◯ Talk loudly from 4 feet
◯ Talk very loudly from 4 feet
◯ Shout from 4 feet
◯ Shout from 2 feet
◯ Shout in ear

Did you ever wear hearing protection (e.g. earplugs or earmuffs) when exposed to engine noise?

◯ Yes
◯ No

What percentage of the time did you wear hearing protection when exposed to the engine noise?

◯ 0% ◯ 10% ◯ 20% ◯ 30% ◯ 40% ◯ 50% ◯ 60% ◯ 70% ◯ 80% ◯ 90%
◯ 100%

These are different types of hearing protection numbered from 1 to 7

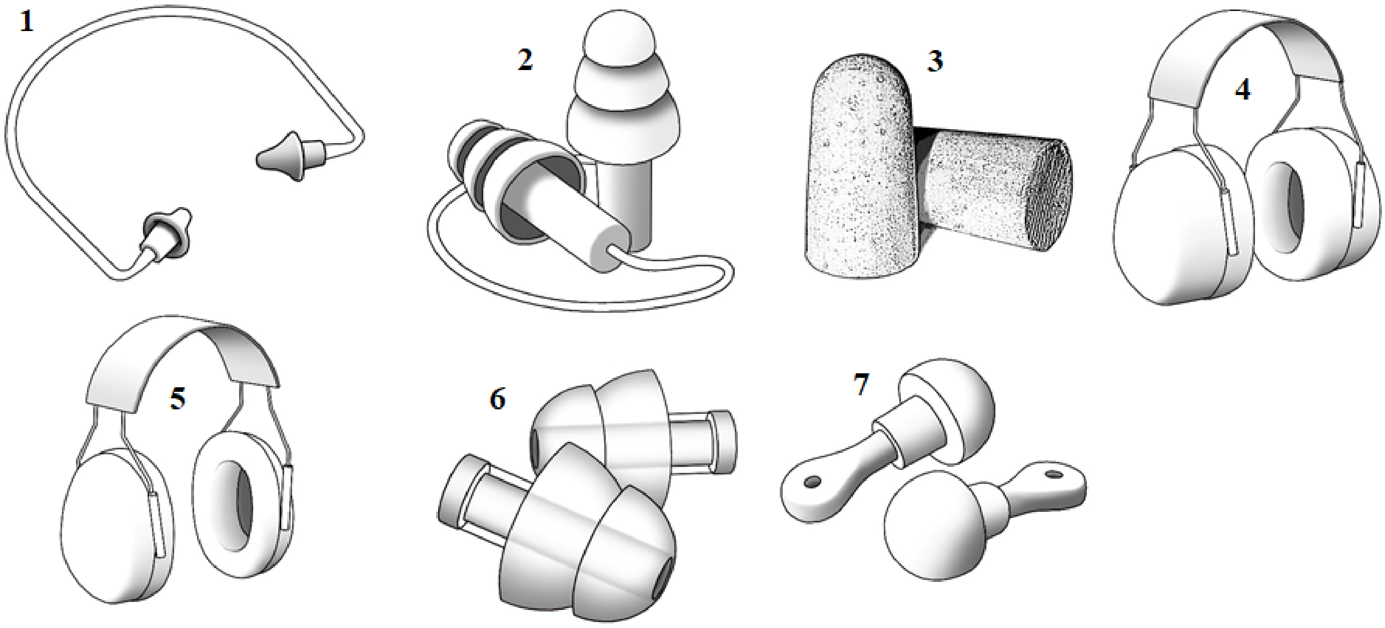

Select the kind of hearing protection you wore

☐ 1 ☐ 2 ☐ 3 ☐ 4
☐ 5 ☐ 6 ☐ 7

In this subsection, we'll ask for more information on your exposure to sport-related noise (e.g. sports matches)

Was the sport-related noise loud enough that you would have needed to raise your voice to communicate with someone 4 feet away?

◯ Yes
◯ No

Tell us approximately how long you were exposed to sport-related noises, by writing the following four numbers on separate lines.

Top line: How many years were you exposed?
Second line: How many weeks per year (on average)?
Third line: How many days per week (on average)?
Fourth line: How many hours per day (on average)?

____

Tell us how loud the sport-related noise was, on average:

If you were trying to communicate with someone 4 feet away (with normal hearing and without hearing protection), how loud would you have to speak?

◯ Talk normally from 4 feet
◯ Raise voice from 4 feet
◯ Talk loudly from 4 feet
◯ Talk very loudly from 4 feet
◯ Shout from 4 feet
◯ Shout from 2 feet
◯ Shout in ear

Did you ever wear hearing protection (e.g. earplugs or earmuffs) when exposed to sport-related noises?

◯ Yes
◯ No

What percentage of the time did you wear the hearing protection when exposed to sport-related noises?

◯ 0% ◯ 10% ◯ 20% ◯ 30% ◯ 40% ◯ 50% ◯ 60% ◯ 70% ◯ 80% ◯ 90%
◯ 100%

These are different types of hearing protection numbered from 1 to 7

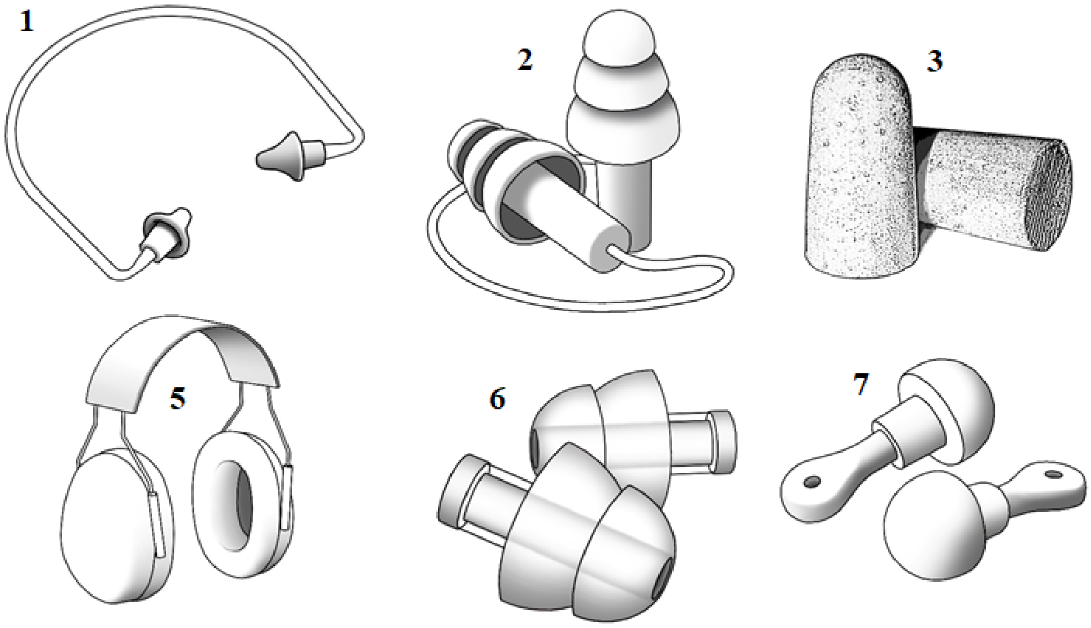

Select the kind of hearing protection you wore when exposed to sport-related noise	☐ 1 ☐ 2 ☐ 3 ☐ 4<br>☐ 5 ☐ 6 ☐ 7

In this subsection, we'll ask for more information on your exposure to noisy cinemas

How loud were the cinemas? Would you have needed to raise your voice to communicate with someone 4 feet away?

◯ Yes
◯ No

In the space available, please specify in 4 lines how long you were exposed to noises in cinemas by writing down in the:

Topline: How many years (approximately)
Second line: How many weeks per year (on average)
Third line: How many days per week (on average)
Fourth line How many hours per day (on average)

____

Tell us how loud the noisy cinemas were, on average:

If you were trying to communicate with someone 4 feet away (with normal hearing and without hearing protection), how loud would you have to speak?

◯ Talk normally from 4 feet
◯ Raise voice from 4 feet
◯ Talk loudly from 4 feet
◯ Talk very loudly from 4 feet
◯ Shout from 4 feet
◯ Shout from 2 feet
◯ Shout in ear

Did you ever wear hearing protection (e.g. earplugs or earmuffs) when exposed to noise from cinemas?

◯ Yes
◯ No

What percentage of the time did you wear the hearing protection when exposed to noises at cinemas?

◯ 0% ◯ 10% ◯ 20% ◯ 30% ◯ 40% ◯ 50% ◯ 60% ◯ 70% ◯ 80% ◯ 90%
◯ 100%

These are different types of hearing protection numbered from 1 to 7

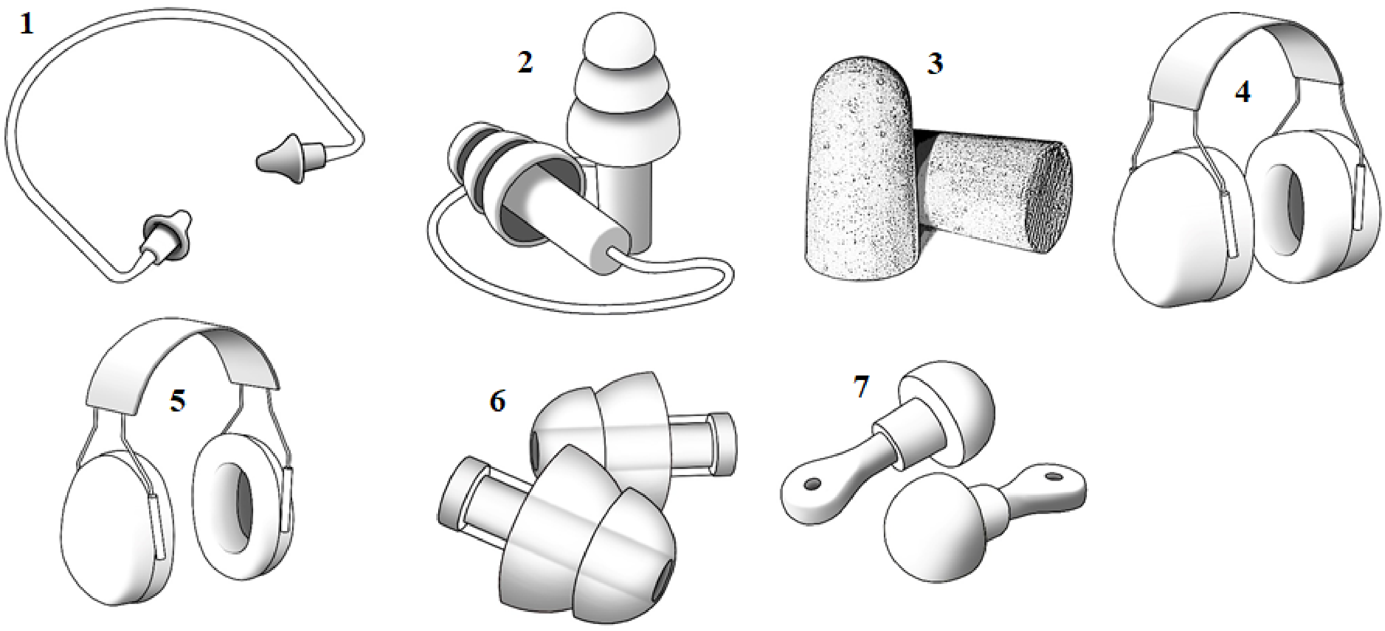

Select the kind of hearing protection you wore in cinemas	☐ 1 ☐ 2 ☐ 3 ☐ 4<br>☐ 5 ☐ 6 ☐ 7

In this subsection, we'll ask for more information on any other recreational/leisure noisy situation

Please specify the recreational / leisure noisy situation/activity	__________________________
Was this other recreational noisy situation loud enough that you would have needed to raise your voice to communicate with someone 4 feet away?	◯ Yes<br>◯ No
Tell us approximately how long you were exposed to the other recreational/leisure noise you specified, by writing the following four numbers on separate lines.<br><br>Top line: How many years were you exposed?<br>Second line: How many weeks per year (on average)?<br>Third line: How many days per week (on average)?<br>Fourth line: How many hours per day (on average)?	________________________________
Tell us how loud this other recreational/leisure noisy situation was, on average:<br><br>If you were trying to communicate with someone 4 feet away (with normal hearing and without hearing protection), how loud would you have to speak?	◯ Talk normally from 4 feet<br>◯ Raise voice from 4 feet<br>◯ Talk loudly from 4 feet<br>◯ Talk very loudly from 4 feet<br>◯ Shout from 4 feet<br>◯ Shout from 2 feet<br>◯ Shout in ear
Did you ever wear hearing protection (e.g. earplugs or earmuffs) when exposed to the noisy situation you've just specified?	◯ Yes<br>◯ No

What percentage of the time did you wear the hearing protection during this other occupational/recreational noisy situation that you specified?

◯ 0% ◯ 10% ◯ 20% ◯ 30% ◯ 40% ◯ 50% ◯ 60% ◯ 70% ◯ 80% ◯ 90%
◯ 100%

These are different types of hearing protection numbered from 1 to 7

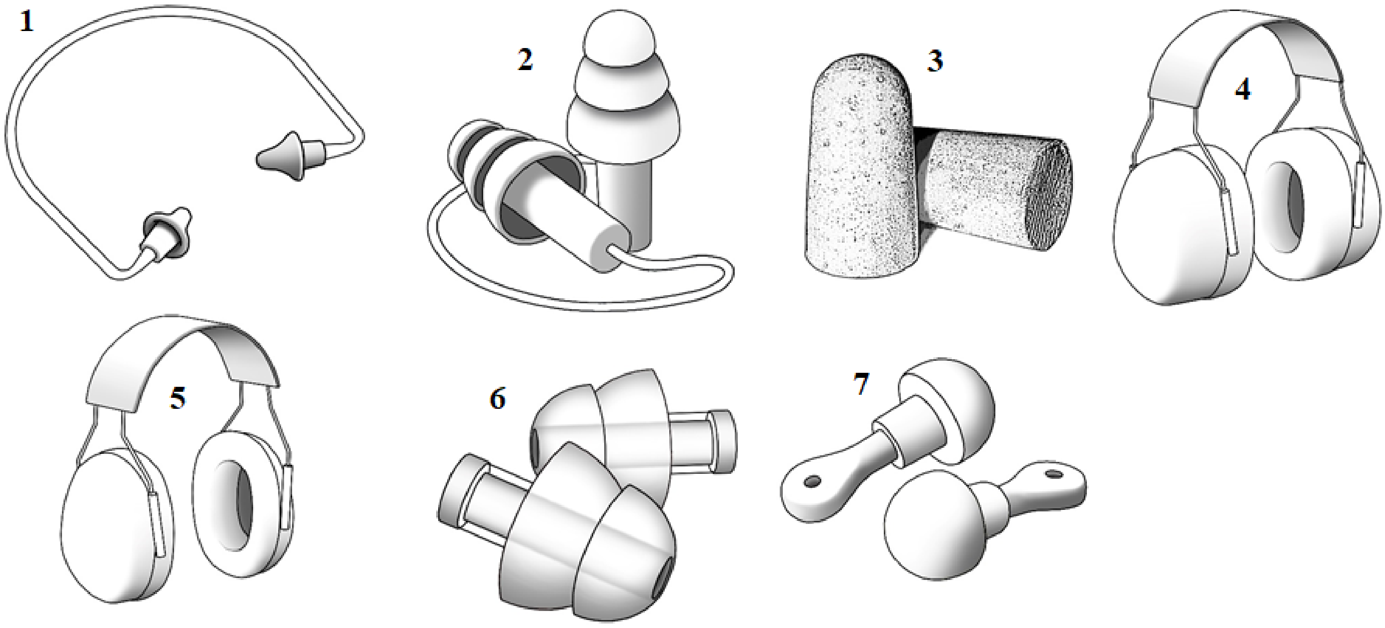

Select the kind of hearing protection you wore when exposed to the other type of recreational / leisure noise	☐ 1 ☐ 2 ☐ 3 ☐ 4<br>☐ 5 ☐ 6 ☐ 7

SECTION C: EARPHONE AND HEADPHONE NOISE EXPOSURE

In this section, we'll ask you to tell us about your use of earphones and headphones.

Have you ever listened through headphones or earphones?	◯ Yes<br>◯ No
Have you ever regularly set the volume control quite high (at or above 70% of maximum volume)?	◯ Yes<br>◯ No
Tell us approximately how long you've been exposed, by writing the following four numbers on separate lines.<br><br>Top line: How many years were you exposed?<br>Second line: How many weeks per year (on average)?<br>Third line: How many days per week (on average)?<br>Fourth line: How many hours per day (on average)?<br><br>ONLY tell use about times when the volume control setting was HIGH (above 70% of maximum volume). Any listening at quieter volumes can be ignored.	____________________________
Now that you've told us how long you've been exposed to noisy earphones/headphones, please specify the average volume-control setting you used during that time (as a percentage of maximum volume).	◯ 70%<br>◯ 75%<br>◯ 80%<br>◯ 85%<br>◯ 90%<br>◯ 95%<br>◯ 100%

SECTION D: Firearm noise exposure

In this section, we'll ask you to tell us about your exposure to firearm noise (gunshots).

We only need to know about exposures WITHOUT HEARING PROTECTION (e.g. earplugs or ear muffs).

Have you ever been exposed to firearm noise (e.g. rifles, handguns) without wearing hearing protection?	◯ Yes<br>◯ No
What type of firearms?	☐ Air gun<br>☐ Low-calibre (.17 or .22) rifle<br>☐ Any other kind of rifle<br>☐ Any other kind of firearm (e.g. handgun, shotgun)
For low-calibre (.17 and .22) rifles, approximately how many rounds have you been exposed to without hearing protection?	__________________________________
For rifles (of higher calibre than .17 or .22), approximately how many rounds have you been exposed to without hearing protection?	__________________________________
For other kinds of firearm (e.g. handguns, shotguns, machine-guns), approximately how many rounds have you been exposed to without hearing protection?	__________________________________

Speech Spatial Qualities SSQ-12

Please complete the Speech Spatial Qualities SSQ-12 Questionnaire

Thank you!

The following 12 questions inquire about aspects of your ability and experience hearing and listening in different situations.

For each question, select one answer choice from the drop-down list. Answer choices run from 0 through to 10. Selecting 10 means that you would be perfectly able to do or experience what is described in the question. Selecting 0 means you would be unable to do or experience what is described. If one question is not applicable to you, select "not applicable" choice.

1. You are talking with one other person and there is a TV on in the same room. Without turning the TV down, can you follow what the person you're talking to says?

○ Not applicable ○ 0 (Not at all) ○ 1 ○ 2 ○ 3 ○ 4 ○ 5 ○ 6 ○ 7 ○ 8
○ 9 ○ 10 (Perfectly)

2. You are listening to someone talking to you, while at the same time trying to follow the news on TV. Can you follow what both people are saying?

○ Not applicable ○ 0 (Not at all) ○ 1 ○ 2 ○ 3 ○ 4 ○ 5 ○ 6 ○ 7 ○ 8
○ 9 ○ 10 (Perfectly)

3. You are in conversation with one person in a room where there are many other people talking. Can you follow what the person you are talking to is saying?

○ Not applicable ○ 0 (Not at all) ○ 1 ○ 2 ○ 3 ○ 4 ○ 5 ○ 6 ○ 7 ○ 8
○ 9 ○ 10 (Perfectly)

4. You are in a group of five people in a busy restaurant. You can see everyone else in the group. Can you follow the conversation?

○ Not applicable ○ 0 (Not at all) ○ 1 ○ 2 ○ 3 ○ 4 ○ 5 ○ 6 ○ 7 ○ 8
○ 9 ○ 10 (Perfectly)

5. You are with a group and the conversation switches from one person to another. Can you easily follow the conversation without missing the start of what each new speaker is saying?

○ Not applicable ○ 0 (Not at all) ○ 1 ○ 2 ○ 3 ○ 4 ○ 5 ○ 6 ○ 7 ○ 8
○ 9 ○ 10 (Perfectly)

6. You are outside. A dog barks loudly. Can you tell immediately where it is, without having to look?

◯ Not applicable ◯ 0 (Not at all) ◯ 1 ◯ 2 ◯ 3 ◯ 4 ◯ 5 ◯ 6 ◯ 7 ◯ 8
◯ 9 ◯ 10 (Perfectly)

7. Can you tell how far away a bus or truck is, from the sound?

◯ Not applicable ◯ 0 (Not at all) ◯ 1 ◯ 2 ◯ 3 ◯ 4 ◯ 5 ◯ 6 ◯ 7 ◯ 8
◯ 9 ◯ 10 (Perfectly)

8. Can you tell from the sound whether a bus or truck is coming towards you or going away?

◯ Not applicable ◯ 0 (Not at all) ◯ 1 ◯ 2 ◯ 3 ◯ 4 ◯ 5 ◯ 6 ◯ 7 ◯ 8
◯ 9 ◯ 10 (Perfectly)

9. When you hear more than one sound at a time, do you have the impression that it seems like a single jumbled sound?

◯ Not applicable ◯ 0 (Jumbled) ◯ 1 ◯ 2 ◯ 3 ◯ 4 ◯ 5 ◯ 6 ◯ 7 ◯ 8
◯ 9 ◯ 10 (Not jumbled)

10. When you listen to music, can you make out which instruments are playing?

◯ Not applicable ◯ 0 (Not at all) ◯ 1 ◯ 2 ◯ 3 ◯ 4 ◯ 5 ◯ 6 ◯ 7 ◯ 8
◯ 9 ◯ 10 (Perfectly)

11. Do every day sounds that you can hear easily seem clear to you (not blurred)?

◯ Not applicable ◯ 0 (Not at all) ◯ 1 ◯ 2 ◯ 3 ◯ 4 ◯ 5 ◯ 6 ◯ 7 ◯ 8
◯ 9 ◯ 10 (Perfectly)

12. Do you have to concentrate very much when listening to someone or something?

◯ Not applicable ◯ 0 (Concentrate hard) ◯ 1 ◯ 2 ◯ 3 ◯ 4 ◯ 5 ◯ 6 ◯ 7
◯ 8 ◯ 9 ◯ 10 (No need to concentrate)

Supplementary Table 1: Original analyses reported in the paper versus analyses excluding participants without noise exposure

	Lifetime noise exposure scores analyses		Effect of lifetime noise exposure on DIN thresholds		Effect of lifetime noise exposure on CRM thresholds		Effect of lifetime noise exposure on SSQ12		Effect of lifetime noise exposure on number of subjects with tinnitus		Effect of lifetime noise exposure on THI		Effect of lifetime noise exposure on hyperacusis scores	
Paper Secti-on	3.1		3.2.1.		3.2.1.		3.3.1.		3.4.1.		3.4.1.		3.5.1.	
	1	2	1	2	1	2	1	2	1	2	1	2	1	2
N	295	267	94 (Y) 47 (O)	86 (Y) 45 (O)	94 (Y) 47 (O)	86 (Y) 45 (O)	217 (Y) 78(O)	196 (Y) 71 (O)	217(Y) 78(O)	196 (Y) 71 (O)	40(Y) 26(O)	40 (Y) 24 (O)	217(Y) 78(O)	196 (Y) 71 (O)
F	-	-	1.85 (Y) 0.71 (O)	0.230 (Y) 0.89 (O)	3.68(Y) 0.815(O)	4.135 (Y) 0.914 (O)	2.21(Y) 1.12(O)	2.435(Y) 0.024(O)	-	-	0.49(Y) 0.49(O)	0.916 (Y) 0.466 (O)	2.81 (Y) 0.93 (O)	10.144 (Y) 2.758 (O)
R^2	-		0.077 (Y) 0.064 (O)	0.095 (Y) 0.082 (O)	0.142(Y) 0.072(O)	0.170 (Y) 0.084 (O)	0.04(Y) 0.059(O)	0.049 (Y) 0.402 (O)	-	-	0.05(Y) 0.09(O)	0.489 (Y) 0.089 (O)	0.05 (Y) 0.049 (O)	0.055 (Y) 0.056 (O)
p-value	0.142	0.173	0.508 (Y) 0.337 (O)	0.663 (Y) 0.479 (O)	0.237(Y) 0.852(O)	0.481 (Y) 0.563 (O)	0.104 (Y) 0.06 (O)	0.015 (Y) 0.274 (O)	0.005 (Y) 0.516 (O)	0.081 (Y) 0.403 (O)	0.307 (Y) 0.461 (O)	0.307 (Y) 0.867 (O)	0.001 (Y) 0.812 (O)	0.002 (Y) 0.956 (O)
OR	-	-	-	-	-	-	-	-	1.50(Y) 1.11(O)	1.34 (Y) 1.20 (O)	-	-	-	-
U	9410	7783.5	-	-	-	-	-		-		-	-	-	-

1: Original analyses reported in paper
2: Analysis including participants with noise exposure only (participants without noise exposure excluded)
Y: Young group
O: Older group

Tinnitus Functional Index

Please complete the Tinnitus Functional Index questionnaire

Thank you!

The purpose of this questionnaire is to identify difficulties that you may be experiencing because of your tinnitus. If you indicate you have tinnitus, please answer all questions.

The word 'tinnitus' refers to the perception of sound in the absence of any corresponding external sound. This noise may be heard in one ear, in both ears or in the middle of the head or it may be difficult to pinpoint its exact location. The noise may be low, medium or high-pitched. There may be a single noise or two or more components. The noise may be continuous or it may come and go.

Do you suffer from tinnitus

◯ Yes ◯ No

Please read each of the 25 questions below carefully. To answer a question, select ONE of the choices that best describe your experience.

1. Because of your tinnitus, is it difficult for you to concentrate?

◯ Yes ◯ Sometimes ◯ No

2. Does the loudness of your tinnitus make it difficult for you to hear people?

◯ Yes ◯ Sometimes ◯ No

3. Does your tinnitus make you angry?

◯ Yes ◯ Sometimes ◯ No

4. Does your tinnitus make you feel confused?

◯ Yes ◯ Sometimes ◯ No

5. Because of your tinnitus, do you feel desperate?

◯ Yes ◯ Sometimes ◯ No

6. Do you complain a great deal about your tinnitus?

◯ Yes ◯ Sometimes ◯ No

7. Because of your tinnitus, do you have trouble falling to sleep at night?

◯ Yes ◯ Sometimes ◯ No

8. Do you feel as though you cannot escape your tinnitus?

◯ Yes ◯ Sometimes ◯ No

9. Does your tinnitus interfere with your ability to enjoy your social activities (such as going out to dinner, to the movies)?

◯ Yes ◯ Sometimes ◯ No

10. Because of your tinnitus, do you feel frustrated?

◯ Yes ◯ Sometimes ◯ No

11. Because of your tinnitus, do you feel that you have a terrible disease?

◯ Yes ◯ Sometimes ◯ No

12. Does your tinnitus make it difficult for you to enjoy life?

◯ Yes ◯ Sometimes ◯ No

13. Does your tinnitus interfere with your job or household responsibilities?

◯ Yes ◯ Sometimes ◯ No

14. Because of your tinnitus, do you find that you are often irritable?

◯ Yes ◯ Sometimes ◯ No

15. Because of your tinnitus, is it difficult for you to read?

◯ Yes ◯ Sometimes ◯ No

16. Does your tinnitus make you upset?

◯ Yes ◯ Sometimes ◯ No

17. Do you feel that your tinnitus problem has placed stress on your relationships with members of your family and friends?

◯ Yes ◯ Sometimes ◯ No

18. Do you find it difficult to focus your attention away from your tinnitus and on other things?

◯ Yes ◯ Sometimes ◯ No

19. Do you feel that you have no control over your tinnitus?

◯ Yes ◯ Sometimes ◯ No

20. Because of your tinnitus, do you often feel tired?

◯ Yes ◯ Sometimes ◯ No

21. Because of your tinnitus, do you feel depressed?

◯ Yes ◯ Sometimes ◯ No

22. Does your tinnitus make you feel anxious?

◯ Yes ◯ Sometimes ◯ No

23. Do you feel that you can no longer cope with your tinnitus?

◯ Yes ◯ Sometimes ◯ No

24. Does your tinnitus get worse when you are under stress?

◯ Yes ◯ Sometimes ◯ No

25. Does your tinnitus make you feel insecure?

◯ Yes ◯ Sometimes ◯ No
